# Protonated oxalyl chloride and the ClCO^+^ cation

**DOI:** 10.1107/S2053229624010714

**Published:** 2024-11-20

**Authors:** Sebastian Steiner, Kristina Djordjevic, Valentin Bockmair, Dirk Hollenwäger, Andreas J. Kornath

**Affiliations:** a Department Chemie, Ludwig-Maximilians Universität, Butenandtstrasse 5-13 (Haus D), D-81377 München, Germany; University of North Texas at Dallas, USA

**Keywords:** oxalyl chloride, chloro­carbonyl cation, superacid, protonation, structure elucidation, crystal structure

## Abstract

The reactions of oxalyl chloride were investigated in the binary superacidic systems HF/SbF_5_ and DF/SbF_5_. *O*-Monoprotonated oxalyl chloride was isolated and represents the first example of a protonated acyl chloride. Diprotonated oxalyl chloride is only stable in solution. Salts of the ClCO^+^ cation were syn­thesized from the reactions of oxalyl chloride or COClF with SbF_5_ in 1,1,1,2-tetra­fluoro­ethane (R-134a, CF_3_CFH_2_). The colourless salts were characterized by low-temperature vibrational spectroscopy, NMR spectroscopy and single-crystal X-ray diffraction. (1,2-Di­chloro-2-oxo­ethyl­idene)oxidanium hexa­fluor­ido­anti­monate(V), [C_2_O(OH)Cl_2_][SbF_6_], crystallizes in the monoclinic space group *P*2_1_ and carbonyl chloride hexa­deca­fluorido­triarsenate(V) [ClCO][Sb_3_F_16_], in the trigonal space group *P*3_1_, with two and three formula units per unit cell, respectively. Monoprotonated oxalyl chloride and the ClCO^+^ cation both display very short C—Cl bonds with a strong double-bond character.

## Abstract 2

The crystal structures of monoprotonated oxalyl chloride and the chloro­carbonyl cation are elucidated for the first time. Both display very short C—Cl bonds with a strong double-bond character.

## Introduction

Oxalyl chloride was first prepared by Fauconnier in 1892 by the reaction of diethyl oxalate and phospho­rus penta­chloride (Fauconnier, 1892 ▸). Nowadays, it is commercially produced by the photochlorination of ethyl­ene carbonate (Pfoertner & Oppenländer, 2012 ▸). Due to its high reactivity, oxalyl chloride is one of the most versatile organic reagents in chemical syn­theses. Among others, it is used in chlorinations, oxidations, reductions, dehydrations, deca­rboxylations or formyl­ation reactions (Masaki & Fukui, 1977 ▸; Omura & Swern, 1978 ▸; Shiri & Kazemi, 2017 ▸; Denton *et al.*, 2012 ▸; Wasserman & Tremper, 1977 ▸; Mendelson & Hayden, 1996 ▸). The best-known applications are its use in Friedel–Crafts reactions (Alexandrou, 1969 ▸; Ketcha & Gribble, 1985 ▸) or the Swern oxidation, *i.e.* the oxidation of primary and secondary alcohols to aldehydes and ketones (Omura & Swern, 1978 ▸).

The reactivity of oxalyl chloride towards strong Lewis acids has been thoroughly investigated. Thus, the chloro­carbonyl cation (ClCO^+^) is observed in the reaction of oxalyl chloride with SbF_5_ (Prakash *et al.*, 1991 ▸). The ClCO^+^ cation is a representative of the com­pound class of linear triatomic mol­ecules, such as OCS, ONP or ONS^+^, for which numerous theoretical calculations have been performed (Peterson *et al.*, 1991 ▸; Pak & Woods, 1997 ▸). It can be synthesized by reacting SbF_5_ with either oxalyl chloride, phosgene or carbonyl chloride fluoride. Furthermore, the reaction of carbon mon­oxide with chlorine in SO_2_ClF/SbF_5_ leads to the formation of the ClCO^+^ cation (Prakash *et al.*, 1991 ▸; Bernhardt *et al.*, 1999 ▸; Christe *et al.*, 1999 ▸). The latter was investigated by Olah in 1991 using NMR spectroscopy (Prakash *et al.*, 1991 ▸), while Aubke characterized the species by Raman and IR spectroscopy for the first time in 1999 (Bernhardt *et al.*, 1999 ▸). However, it has not yet been possible to elucidate the crystal structure of the cation due to its high reactivity. This prompted us to isolate the ClCO^+^ cation and to perform single-crystal X-ray diffraction analysis to structurally characterize the cation and to com­pare its bond lengths with isoelectronic mol­ecules such as OCS, which shows a short C—S bond with a strong C=S double-bond character (Pak & Woods, 1997 ▸).

Furthermore, in previous studies by our group, mono- and diprotonated species of oxalic acid, pyruvic acid and parabanic acid were isolated and characterized, whereas the dications constitute vicinal superelectrophiles (Schickinger *et al.*, 2018 ▸; Virmani *et al.*, 2022 ▸; Beck *et al.*, 2020 ▸). In addition, we recently investigated the reactivity of haloacetyl fluorides in superacidic media and observed the protonation of the carbonyl bond, as well as HF addition to the latter, as in the case of di­chloro­acetyl fluoride (Steiner *et al.*, 2022 ▸, 2024 ▸). This prompt­ed us to perform investigations on the reactivity of oxalyl chloride in the superacidic system HF/SbF_5_.<!?tpb=-20pt>

## Results and discussion

### Syntheses and properties of [C_2_O(O*X*)Cl_2_][SbF_6_] (*X* = H or D), [C_2_(OH)_2_Cl_2_][Sb_*n*_F_5*n*+1_]_2_, [ClCO][Sb_3_F_15_Cl] and [ClCO][Sb_3_F_16_]

Oxalyl chloride was reacted in the binary superacidic systems HF/SbF_5_ and DF/SbF_5_. According to reaction (1) in Scheme 1, [C_2_O(OH)Cl_2_][SbF_6_] (**1**) and [C_2_O(OD)Cl_2_][SbF_6_] (**2**) were obtained as *O*-monoprotonated species of oxalyl chloride in qu­anti­tative yields as colourless solids.
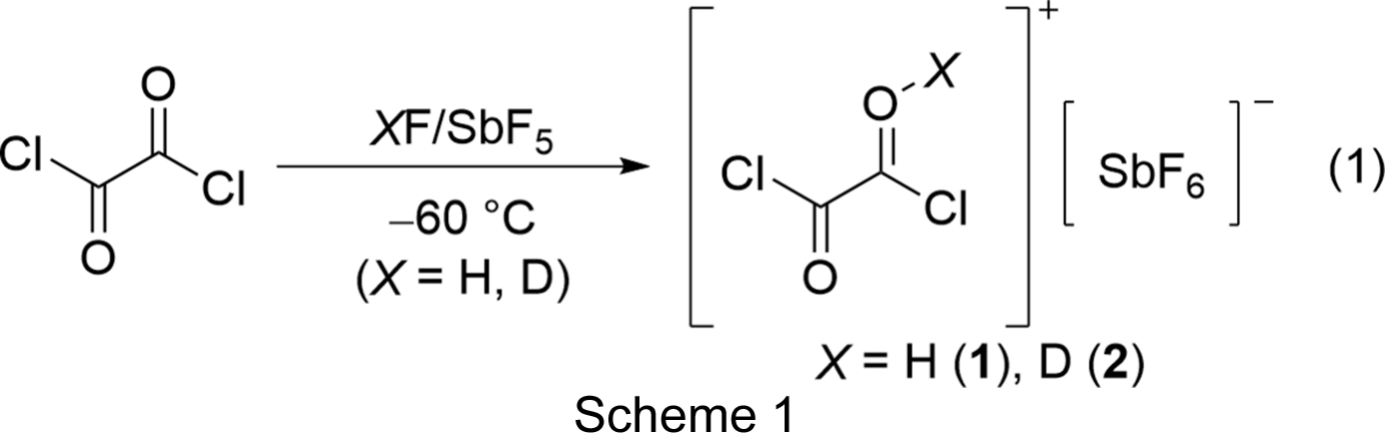

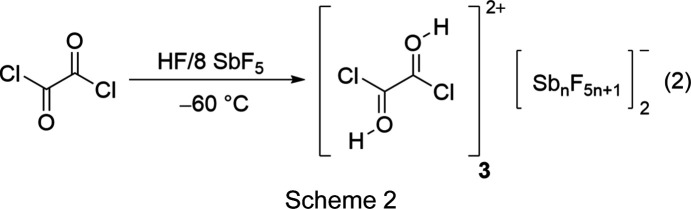


To obtain the diprotonated species, oxalyl chloride was reacted in the binary superacidic system HF/SbF_5_ with an excess of the strong Lewis acid SbF_5_. However, even with a tenfold excess of SbF_5_, the isolation of diprotonated oxalyl chloride (**3**) as a solid was not possible. Instead of the desired species, monoprotonated oxalyl chloride (**1**) was obtained. To investigate the reaction of oxalyl chloride in anhydrous hy­dro­gen fluoride (aHF) at −60 °C with an excess of SbF_5_, NMR spectroscopy was performed employing eight equivalents of SbF_5_. Accordingly, the measured ^1^H, ^19^F and ^13^C NMR spectra indicate the presence of **3** in the solution, as pre­sent­ed in reaction (2) in Scheme 2. Salts **1** and **2** show thermal decom­position at −45 °C.

[ClCO][Sb_3_F_15_Cl] (**4**) was synthesized by reacting oxalyl chloride with three equivalents of SbF_5_ in 1,1,1,2-tetra­fluoro­ethane (R-134a, CF_3_CFH_2_) at −78 °C. **4** was obtained as a colourless solid according to reaction (3) in Scheme 3. To avoid mixed occupancies of the fluorine positions of the anion with chlorine, carbonyl chloride fluoride was reacted under the same conditions to form [ClCO][Sb_3_F_16_] (**5**), as pre­sent­ed in reaction (4) in Scheme 3 (Prakash *et al.*, 1991 ▸; Bernhardt *et al.*, 1999 ▸; Christe *et al.*, 1999 ▸).
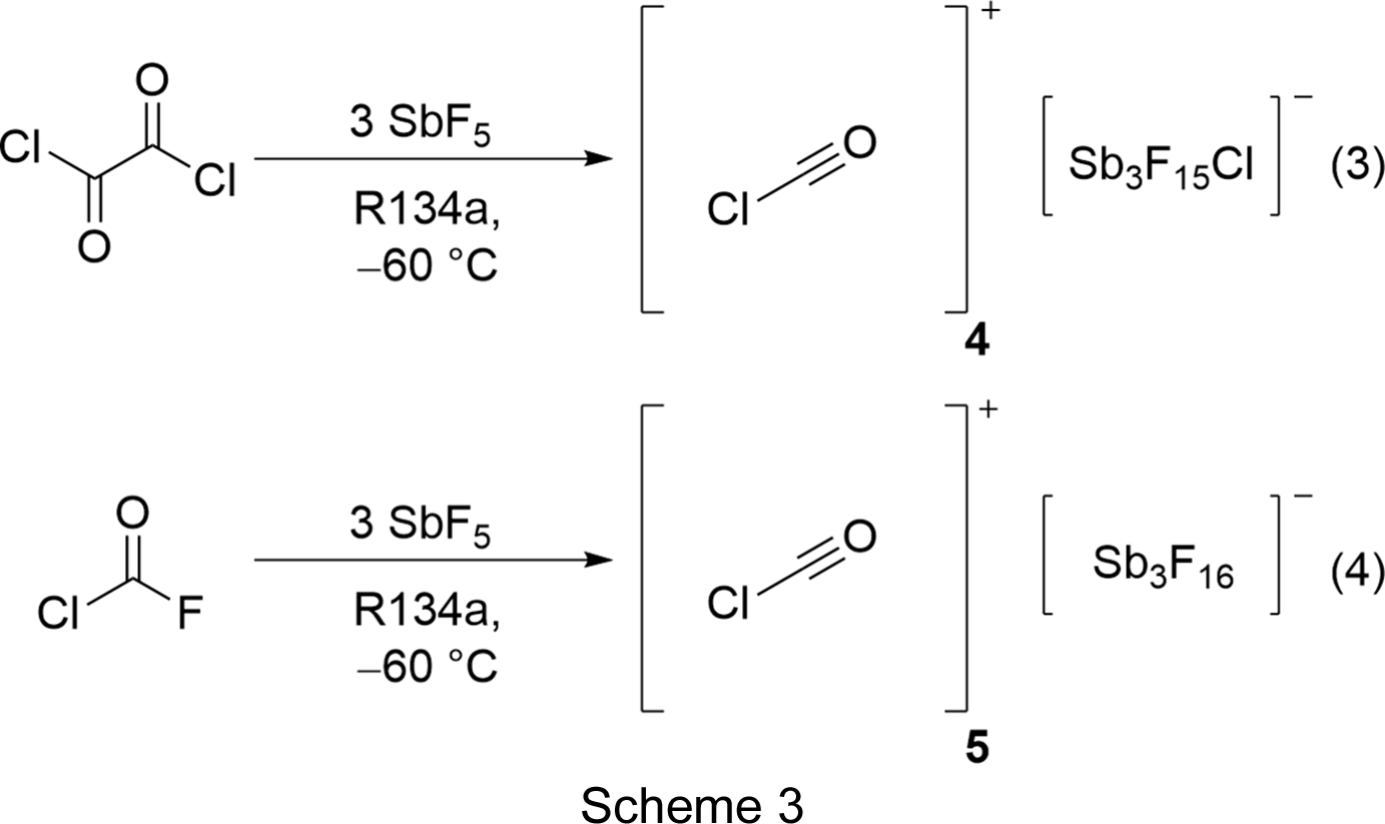


### Vibrational spectroscopy

**Figure 1:**
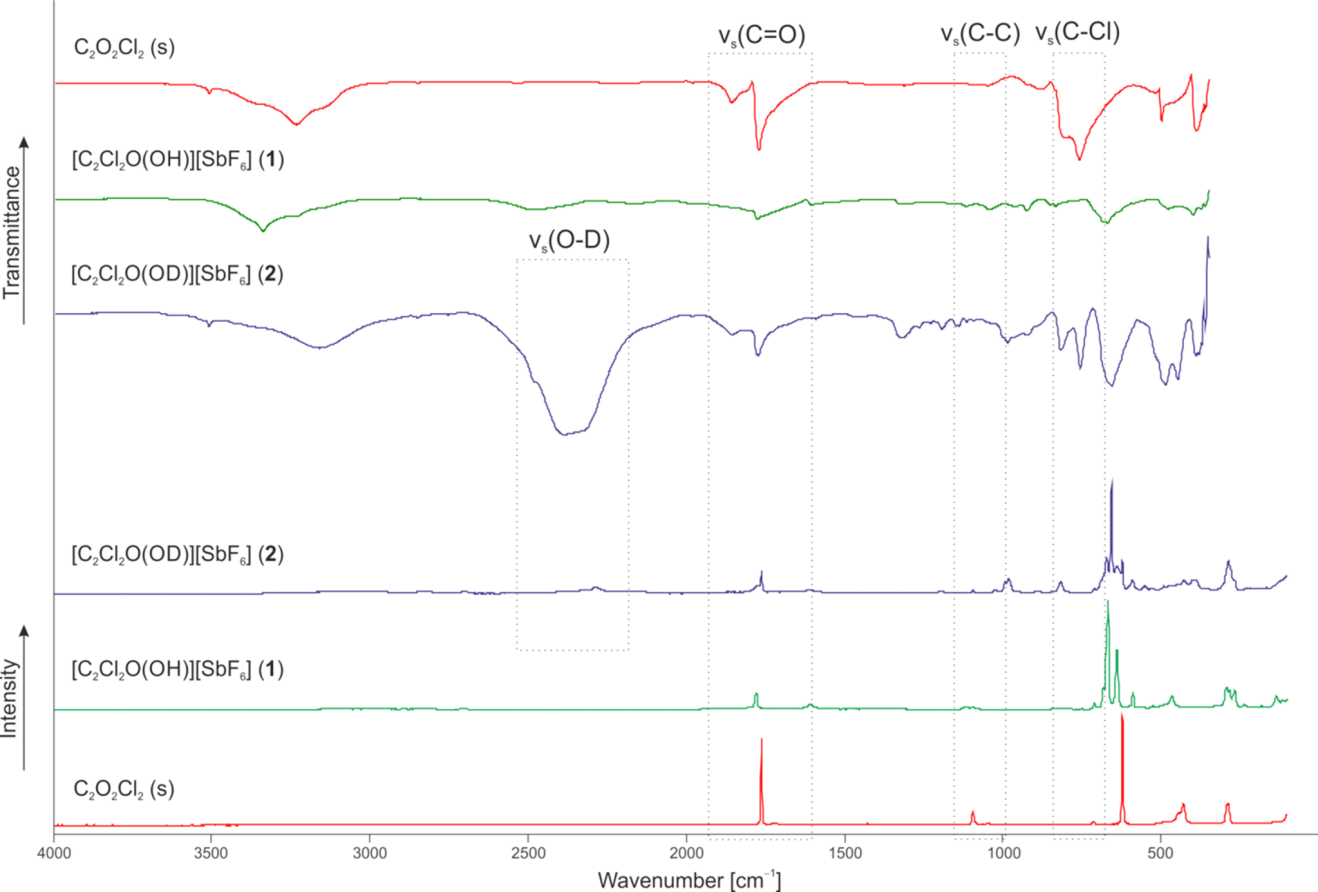
Low-temperature Raman (bottom) and IR spectra (top) of [C_2_O(O*X*)Cl_2_][SbF_6_] (**1** and **2**) (*X* = H or D) and C_2_O_2_Cl_2_.

The low-temperature Raman (Ra) and infrared (IR) spectra of [C_2_O(OH)Cl_2_][SbF_6_] (**1**), [C_2_O(OD)Cl_2_][SbF_6_] (**2**) and C_2_O_2_Cl_2_ are illustrated in Fig. 1 ▸. The com­plete vibrational frequencies of **1**, **2**, **4** and **5**, as well as of oxalyl chloride and carbonyl chloride fluoride, are provided in the supporting information (see Figs. S1–S3 and Tables S1–S5) (Bernhardt *et al.*, 1999 ▸; Davis *et al.*, 1993 ▸; Nielsen *et al.*, 1952 ▸).

For the [C_2_O(OH)Cl_2_]^+^ cation with *C_s_* symmetry, 15 fun­da­mental vibrational modes are expected, all of which are Raman and IR active. ν_s_(O—H) is superposed by con­den­sed water in the IR spectra due to the measuring method. Furthermore, the O—H stretching vibration shows low intensity in the Raman spectra due to the poor polarizability of the O—H group, which does not apply to the O—D group. The O—D stretching vibration of the d-isotopomeric species **2** is observed at 2157 cm^−1^ in the Raman spec­trum and at 2388 cm^−1^ in the IR spectrum. The C=O stretching vibration of the protonated COCl moiety is de­tected at 1607 cm^−1^ (Ra) (**1** and **2**), as well as at 1605 (**1**) and 1593 cm^−1^ (**2**) (IR), and is significantly red-shifted (by ap­prox­imately 165 cm^−1^) com­pared to the starting material. The vibrations are red-shifted by around 100–150 cm^−1^ in com­parison to the corresponding vibrations of protonated acyl fluorides (Steiner *et al.*, 2022 ▸, 2024 ▸; Bayer *et al.*, 2022 ▸). The ν_s_(C=O) of the adjacent unprotonated carbonyl group is not affected by the protonation. The C—Cl stretching vibration of the protonated COCl moiety appears at 817 cm^−1^ (**2**) in the Raman spectrum and at 833 cm^−1^ (**1**), as well as at 818 cm^−1^ (**2**), in the IR spectrum, *i.e.* blue-shifted by approximately 70 cm^−1^. The ν_s_(C—Cl) of the neighboring COCl moiety is not affected by protonation and occurs at 623 cm^−1^ (**2**) in the Raman spectrum. Additionally, the C—C stretching vibration is detected at 1118 (Ra) (**1**) and 1117 cm^−1^ (**1** and **2**) (IR), and is thus blue-shifted by approximately 20 cm^−1^ in com­parison to oxalyl chloride.

More vibrations are observed for the [SbF_6_]^−^ anion than expected for ideal octa­hedral symmetry due to inter­ionic inter­actions in the solid leading to a symmetry distortion (Weidlein *et al.*, 1988 ▸).

### Crystal structure of [C_2_O(OH)Cl_2_][SbF_6_] (1)

**Figure 2:**
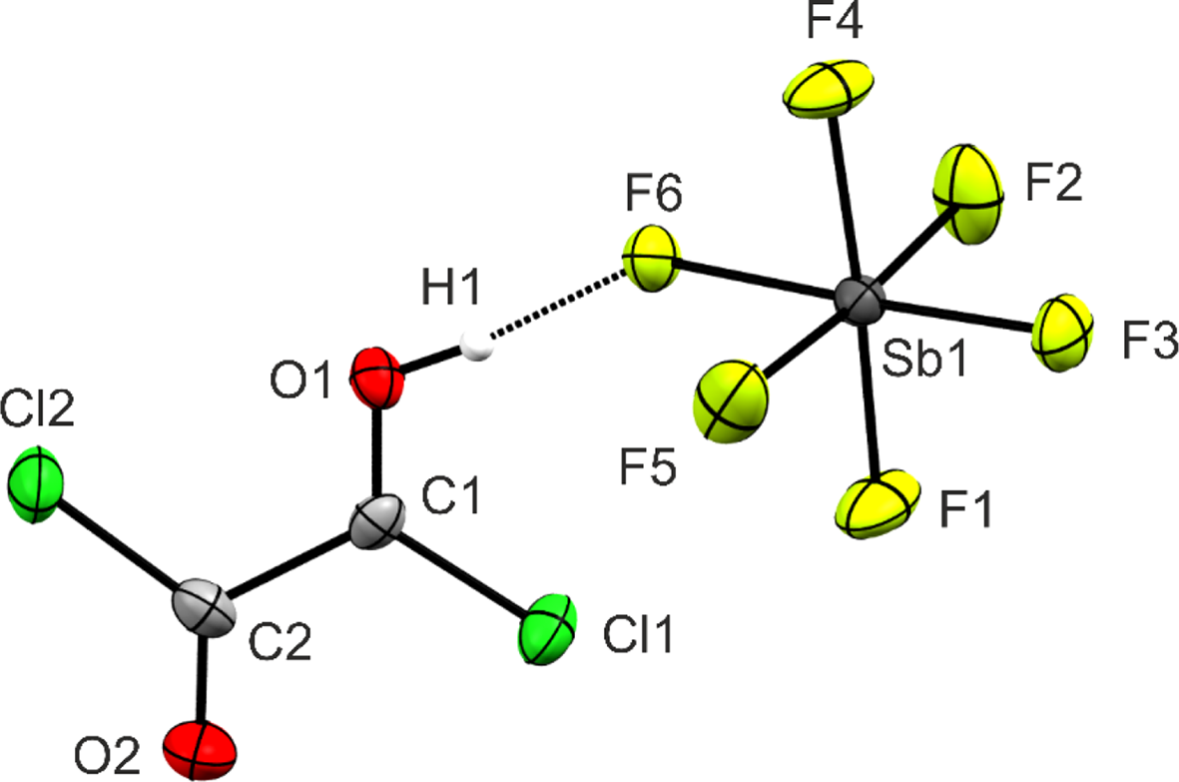
The asymmetric unit of **1**, with displacement ellipsoids drawn at the 50% probability level.

The hexa­fluorido­anti­monate of monoprotonated oxalyl chlo­ride (**1**) crystallizes in the monoclinic space group *P*2_1_ with two formula units per unit cell. The asymmetric unit is illustrated in Fig. 2 ▸. Crystal data and structure refinement details are provided in Table 1 ▸ and in the supporting information (Tables S6 and S7).

Due to the protonation, the C1—O1 bond length [1.225 (8) Å] is significantly elongated com­pared to the starting material [1.180 (2) Å; Danielson *et al.*, 1995 ▸] and is longer than a formal C=O bond (1.18 Å; Allen *et al.*, 1987 ▸). The C2—O2 bond length [1.184 (8) Å] of the adjacent un­pro­tonated C=O bond is not affected by the protonation [1.180 (2) Å; Danielson *et al.*, 1995 ▸]. Furthermore, the C1—Cl1 bond length [1.647 (7) Å] is significantly shortened com­pared to the neutral com­pound [1.747 (3) Å; Danielson *et al.*, 1995 ▸] and is in the range between a formal C—Cl single bond (1.76 Å; Allen *et al.*, 1987 ▸) and a C=Cl double bond (1.56 Å; Holleman *et al.*, 1987 ▸). The same applies for the C2—Cl2 bond length [1.693 (7) Å] [*cf.* 1.747 (3) Å for oxalyl chloride; Danielson *et al.*, 1995 ▸]. The elongation of the C=O bond and the shortening of the C—Cl bonds are consistent with the observed shifts of the ν_s_(C=O) and ν_s_(C—Cl) in the vibrational spectra. The C1—C2 bond length [1.550 (8) Å] is not affected by protonation [*cf.* 1.545 (8) Å for oxalyl chloride; Danielson *et al.*, 1995 ▸]. This is consistent with the results ob­served for the mono- and diprotonated species of oxalic acid. In both cases, the protonation at the carbonyl groups does not affect the bond lengths of the C—C backbone (Schickinger *et al.*, 2018 ▸).

The Sb—F bond lengths are in the range between 1.846 (4) and 1.941 (3) Å, and correspond with values reported in the literature (Minkwitz *et al.*, 1999*a* ▸,*b* ▸; Minkwitz & Schneider, 1999 ▸). Due to inter­ionic inter­actions, the anion displays dis­torted octa­hedral symmetry. The Sb1—F6 bond [1.941 (3) Å] is significantly longer than the other Sb—F bonds with it being involved in hy­dro­gen bonding.

In the crystal structure of **1**, the ions are arranged into chains along the *a* and *b* axes by the strong O1(—H1)⋯F6 hy­dro­gen bond [2.421 (7) Å] and the C⋯F inter­actions C1⋯F2^i^ [2.617 (7) Å; see Fig. 4 for symmetry codes] and C1⋯F4^ii^ [2.565 (7) Å] (see Figs. S4–S5) (Jeffrey, 1997 ▸; Bondi, 1964 ▸). The chains are linked to each other by the Cl⋯F inter­action Cl1⋯F3^iii^ [2.883 (5) Å] to form layers. All inter­atomic C⋯F and Cl⋯F contacts are below the sum of the van der Waals radii (3.17 and 3.22 Å; Bondi, 1964 ▸). Inter­atomic distances are listed in Table S7.

### Crystal structure of [ClCO][Sb_3_F_16_] (5)

The crystal structures of **4** and **5** were both obtained by recrystallizing the salts from R-134a (CF_3_CFH_2_) at −40 °C. As **4** crystallizes as an inversion twin and shows mixed occupancies of all 16 crystallographic fluorine positions with chlorine, the structural parameters show high standard deviations. Therefore, the crystal structure of **5** is used for the discussion of all experimental parameters.

**Figure 3:**
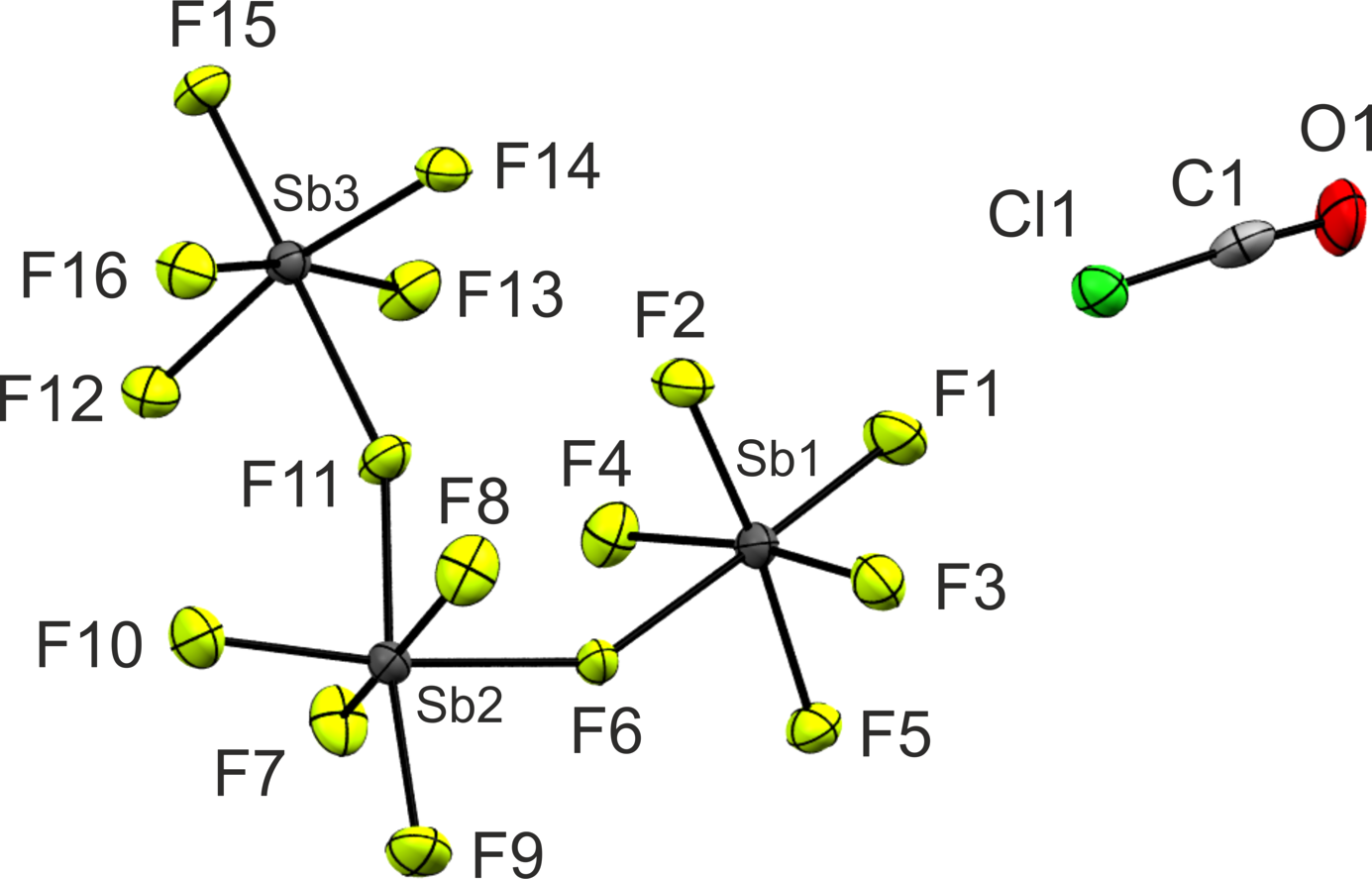
The asymmetric unit of **5**, with displacement ellipsoids drawn at the 50% probability level.

[ClCO][Sb_3_F_16_] (**5**) crystallizes in the trigonal space group *P*3_1_ with three formula units per unit cell. The asymmetric unit is depicted in Fig. 3 ▸. Crystal data and structure refinement details are provided in Table 1 ▸ and in the supporting information (Tables S6 and S8).

The C1—O1 bond length [1.105 (10) Å] is significantly shortened com­pared to ClFCO [1.173 (2) Å; Oberhammer, 1980 ▸]. It is in the range between a formal C=O double bond (1.19 Å) and a C≡O triple bond (1.07 Å) (Allen *et al.*, 1987 ▸). It corresponds with the C=O bond length (1.1562 Å) of the isoelectronic mol­ecule OCS (Pak & Woods, 1997 ▸). Furthermore, the C1—Cl1 bond length [1.571 (3) Å] is significantly shortened com­pared to ClFCO [1.725 (2) Å; Oberhammer, 1980 ▸]. It is in the range of a formal C=Cl double-bond length (1.56 Å) and thus displays a significant double-bond character (Allen *et al.*, 1987 ▸). It corresponds with the C—Cl bond length [1.67 (2) Å] of ClCN (Beach & Turkevich, 1939 ▸). The Cl1—C1—O1 angle [176.1 (8)°] indicates the essentially linear structure of the cation.

The Sb—F bond lengths of the terminal F atoms are in the range between 1.831 (8) and 1.857 (7) Å. The bridging Sb—F bonds are longer than the terminal ones, with bond lengths up to 2.088 (8) Å. The bond angles Sb1—F6—Sb2 [146.0 (4)°] and Sb2—F11—Sb3 [156.6 (4)°] are also in good agreement with the literature (Faggiani *et al.*, 1986 ▸; Gerken *et al.*, 2002 ▸).

**Figure 4:**
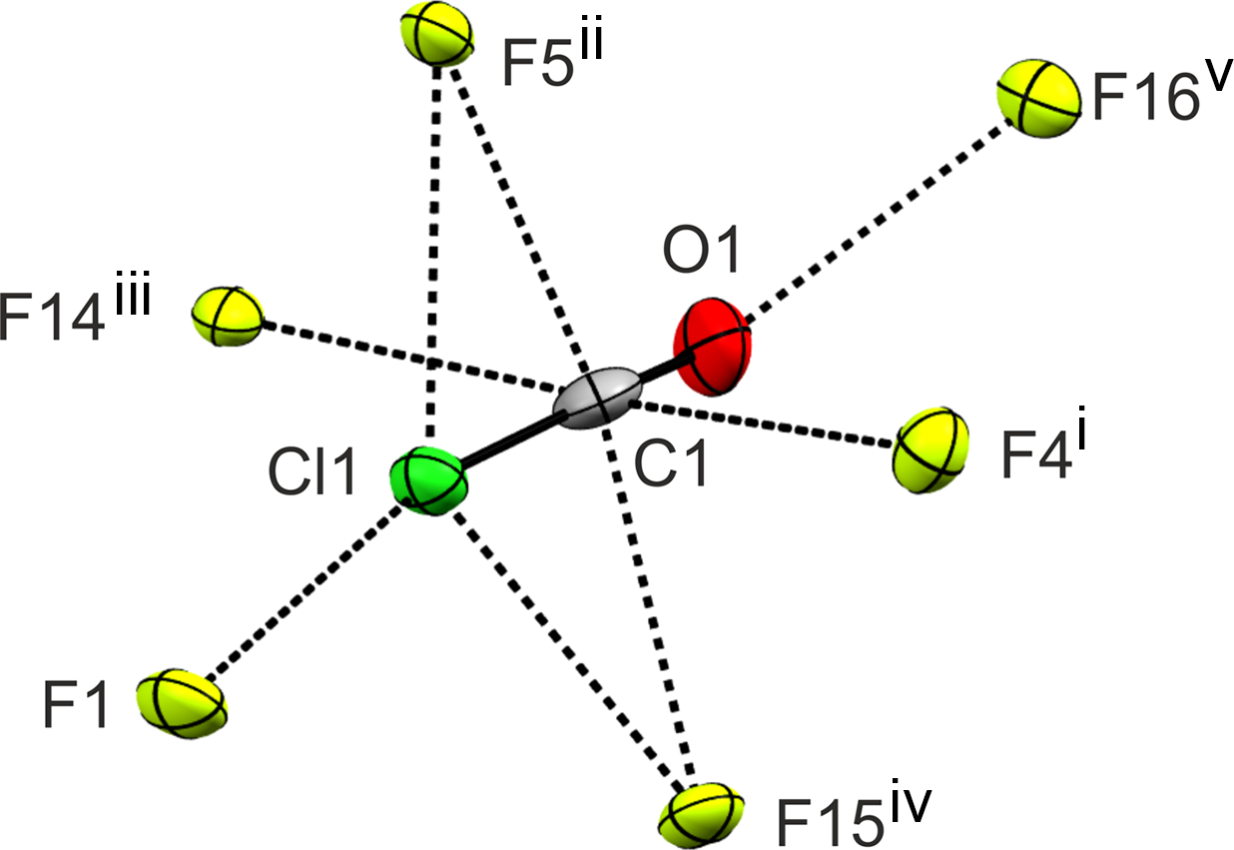
Inter­atomic contacts of **5**, with displacement ellipsoids drawn at the 50% probability level. [Symmetry codes: (i) *x* − 1, *y*, *z*; (ii) −*x* + *y*, −*x* + 1, *z* − 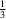; (iii) *x*, *y* + 1, *z*; (iv) −*y*, *x* − *y*, *z* + 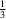; (v) *x* − 1, *y* + 1, *z*.

In the crystal structure of **5**, the ions form a helical structure along the *c* axis *via* the C⋯F inter­action C1⋯F5^ii^ (2.75 Å) and the Cl⋯F inter­action Cl1⋯F1 [2.56 (1) Å] (see Fig. 4 ▸, and Figs. S6–S7 in the supporting information). The cation displays a tetra­coordinated C1 atom and further forms the inter­ionic contacts C1⋯F4^i^ (2.968 Å), C1⋯F14^iii^ (3.062 Å), C1⋯F15^iv^ (2.92 Å) and O1⋯F16^v^ [2.79 (2) Å] (see Fig. 4 ▸). All inter­atomic C⋯F, Cl⋯F and O⋯F contacts are below the sum of the van der Waals radii (3.17, 3.22 and 2.99 Å; Bondi, 1964 ▸). Selected inter­atomic distances are listed in Table S8.

### NMR spectroscopy

To trace the reactivity of oxalyl chloride in aHF and the binary superacidic system HF/SbF_5_, the ^1^H, ^19^F and ^13^C NMR spectra were measured at −60 °C with acetone-*d*_6_ as the external standard. The measured NMR spectra and the com­plete NMR data of oxalyl chloride, **1** and **3** are listed in the supporting information (Figs. S8–S18).

The ^13^C NMR spectrum of oxalyl chloride dissolved in aHF at −60 °C shows a singlet at 161.1 ppm for both COCl moieties. Furthermore, no chlorine–fluorine exchange or HF addition to the carbonyl bond is observed under these con­ditions.

By first dissolving equimolar amounts of SbF_5_ com­pared to oxalyl chloride in HF and then adding the acyl chloride, monoprotonated oxalyl chloride (**1**) is formed. The ^1^H NMR spectrum shows a singlet at 10.03 ppm for the protonated carbonyl group. The ^13^C NMR spectrum displays two singlets located at 161.7 and 183.4 ppm. These are assigned to the carbonyl groups, whereas the NMR resonance of the protonated COCl moiety is significantly shifted downfield. The ^19^F NMR spectrum indicates chlorine–fluorine exchange initiated by the protonation of oxalyl chloride. Thus, the NMR resonance at 17.74 ppm indicates the formation of oxalyl fluoride. The ^1^H, ^19^F and ^13^C NMR spectra of (COF)_2_ at −60 °C in aHF are illustrated in Figs. S10–S11 (see supporting information). Furthermore, in the ^19^F NMR spectrum, the resonance at −124.78 ppm is assigned to the [SbF_6_]^−^ anion (Dean & Gil­lespie, 1969 ▸).

Since the diprotonated oxalyl chloride (**3**) could not be isolated as a solid, the reaction of oxalyl chloride in HF/SbF_5_ at −60 °C was investigated using NMR spectroscopy, whereas an eightfold amount of SbF_5_ was applied. Accordingly, the NMR spectra indicate the presence of **3** in the solution. The ^1^H NMR spectrum shows a singlet at 9.61 ppm for the protonated COCl^+^ moieties, whereas the ^13^C NMR spectrum displays a singlet at 183.1 ppm. Compared to the neutral com­pound, the ^13^C NMR resonance is significantly shifted downfield. Furthermore, in the ^19^F NMR spectrum, multiple resonances located in the range between −117.46 and −143.05 ppm are assigned to the [Sb_*n*_F_5*n*+1_]^−^ polyanions (Dean & Gillespie, 1969 ▸). Thus, di­pro­tonated oxalyl chloride is stable in solution at −60 °C. However, after removal of the excess HF at −78 °C, it decom­poses with the formation of **1**. Furthermore, as in the NMR spectra of **1**, a chlorine–fluorine exchange is observed, as the signal at 17.46 ppm indicates the formation of oxalyl fluoride. Additional signals in the ^19^F NMR spectra of **3** at −21.78 and −59.90 ppm are assigned to COF_2_ and CF_3_OH (Christe *et al.*, 2007 ▸). The ^1^H, ^19^F and ^13^C NMR spectra of COF_2_ in aHF at −60 °C are depicted in the supporting information (Fig. S12). COF_2_ is likely formed due to the decom­position of oxalyl fluoride under superacidic conditions.

## Conclusions

Oxalyl chloride was reacted in the binary superacidic systems HF/SbF_5_ and DF/SbF_5_ to form the *O*-monoprotonated and its D-isotopomeric species as hexa­fluorido­anti­monates. Both represent the first examples of protonated acyl chlorides. When the Lewis acid SbF_5_ is applied in eightfold excess, di­pro­tonated oxalyl chloride is formed, which is only stable in solution. By the reaction of oxalyl chloride or carbonyl chloride fluoride in the aprotic solvent 1,1,1,2-tetra­fluoro­ethane (R-134a, CF_3_CFH_2_) with a threefold excess of SbF_5_, salts of the chloro­carbonyl cation were isolated. The colourless salts were characterized by low-temperature vibrational spectroscopy and low-temperature NMR spectroscopy. The crystal structures of [C_2_O(OH)Cl_2_][SbF_6_] and [ClCO][Sb_3_F_16_] were determined by single-crystal X-ray diffraction analysis. Monoprotonated oxalyl chloride and the chloro­carbonyl cation both display very short C—Cl bonds with strong dou­ble-bond character.

## Supplementary Material

Crystal structure: contains datablock(s) I, 5, global. DOI: 10.1107/S2053229624010714/yd3050sup1.cif

Structure factors: contains datablock(s) 1. DOI: 10.1107/S2053229624010714/yd30501sup2.hkl

Structure factors: contains datablock(s) 5. DOI: 10.1107/S2053229624010714/yd30505sup3.hkl

Supporting information file. DOI: 10.1107/S2053229624010714/yd3050sup4.pdf

CCDC references: 2304793, 2304777

## Figures and Tables

**Table 1:** Experimental details Experiments were carried out with Mo *K*α radiation using a Rigaku Xcalibur Sapphire3 diffractometer. Absorption was corrected for by multi-scan methods (*CrysAlis PRO*; Rigaku OD, 2020 ▸).

	**1**	**5**
Crystal data		
Chemical formula	(C_2_HCl_2_O_2_)[SbF_6_]	(CClO)[Sb_3_F_16_]
*M*_r_	363.68	732.71
Crystal system, space group	Monoclinic, *P*2_1_	Trigonal, *P*3_1_
Temperature (K)	106	102
*a*, *b*, *c* (Å)	6.1616 (8), 10.8379 (10), 6.8805 (8)	8.0824 (3), 8.0824 (3), 18.3341 (8)
α, β, γ (°)	90, 106.472 (13), 90	90, 90, 120
*V* (Å^3^)	440.61 (9)	1037.22 (9)
*Z*	2	3
μ (mm^−1^)	3.80	6.19
Crystal size (mm)	0.31 × 0.17 × 0.12	0.19 × 0.14 × 0.11

Data collection		
*T*_min_, *T*_max_	0.737, 1.000	0.882, 1.000
No. of measured, independent and observed [*I* > 2σ(*I*)] reflections	8919, 2930, 2692	6310, 3334, 3029
*R*_int_	0.042	0.044
(sin θ/λ)_max_ (Å^−1^)	0.755	0.746

Refinement		
*R*[*F*^2^ > 2σ(*F*^2^)], *wR*(*F*^2^), *S*	0.030, 0.057, 1.04	0.038, 0.077, 1.03
No. of reflections	2930	3334
No. of parameters	122	199
No. of restraints	2	0
H-atom treatment	Only H-atom coordinates refined	–
Δρ_max_, Δρ_min_ (e Å^−3^)	1.18, −0.66	1.36, −1.09
Absolute structure	Refined as an inversion twin	Twinning involves inversion, so Flack parameter cannot be determined
Absolute structure parameter	0.52 (3)	–

Computer programs: *CrysAlis PRO* (Rigaku OD, 2020 ▸), *SHELXT* (Sheldrick, 2015*a* ▸), *SHELXL2019* (Sheldrick, 2015*b* ▸), *ORTEP-3 for Windows* (Farrugia, 2012 ▸) and *PLATON* (Spek, 2020 ▸).
