# Supplementary material for: Protonated oxalyl chloride and the ClCO+ cation
Source: Acta Crystallogr C Struct Chem. 2024 Nov 20;80(Pt 12):792–7. doi: 10.1107/S2053229624010714 (PMC11619778; doi:10.1107/S2053229624010714)
Supplement: Supplementary file 4 [file c-80-00792-sup4.pdf]

STRUCTURAL CHEMISTRY

Volume 80 (2024)

Supporting information for article:

Protonated oxalyl chloride and the ClCO^{+} cation

Sebastian Steiner, Kristina Djordjevic, Valentin Bockmair, Dirk Hollenwäger and Andreas J. Kornath

Supporting Information

Protonated Oxalyl Chloride and the $ClCO^+$ Cation

Sebastian Steiner,* Kristina Djordjevic, Dirk Hollenwäger, Valentin Bockmair and Andreas J. Kornath

Abstract: The reactions of oxalyl chloride were investigated in the binary superacid systems HF/SbF_5 and DF/SbF_5. *O*-monoprotonated oxalyl chloride was isolated and represents the first example of a protonated acyl chloride. Diprotonated oxalyl chloride is only stable in solution. Salts of the $ClCO^+$ cation were synthesized from the reactions of oxalyl chloride or COClF with SbF_5 in R-134a (CF_3CFH_2). The colorless salts were characterized by low-temperature vibrational spectroscopy, low-temperature NMR spectroscopy, and single-crystal X-ray diffraction. $[C_2O(OH)Cl_2][SbF_6]$ crystallizes in the monoclinic space group $P2_1$, $[ClCO][Sb_3F_{16}]$ in the trigonal space group $P3_1$ with two and three formula units per unit cell each. Monoprotonated oxalyl chloride and the $ClCO^+$ cation both display very short C–Cl bonds with a strong double bond character.

Figures:

Figure S1. Low-temperature Raman and IR spectra of $[C_2O(OH)Cl_2][SbF_6]$ (**1**), $[C_2O(OD)Cl_2][SbF_6]$ (**2**) and $C_2O_2Cl_2$.
Figure S2. Low-temperature Raman spectra of $[ClCO][Sb_3F_{15}Cl]$ (**4**) and $C_2O_2Cl_2$.
Figure S3. Low-temperature Raman spectra of $[ClCO][Sb_3F_{16}]$ (**5**) and ClFCO.
Figure S4. Chains along the *a*-axis of **1** (displacement ellipsoids with 50% probability).
Figure S5. Chains along the *b*-axis of **1** (displacement ellipsoids with 50% probability).
Figure S6. Chains along the *c*-axis of **5** (displacement ellipsoids with 50% probability).
Figure S7. Helical structure along the *c*-axis of **5** (displacement ellipsoids with 50% probability).
Figure S8. ^1H and ^{19}F NMR spectra of $C_2O_2Cl_2$ in aHF, −60 °C.
Figure S9. ^{13}C NMR spectrum of $C_2O_2Cl_2$ in aHF, −60 °C.
Figure S10. ^1H and ^{19}F NMR spectra of $C_2O_2F_2$ in aHF, −60 °C.
Figure S11. ^{13}C NMR spectrum of $C_2O_2F_2$ in aHF, −60 °C.
Figure S12. ^1H and ^{19}F NMR spectra of COF_2 in aHF, −60 °C.
Figure S13. ^1H and ^{19}F NMR spectra of $C_2O_2Cl_2$ in HF/SbF_5, −60 °C.
Figure S14. ^{13}C NMR spectrum of $C_2O_2Cl_2$ in HF/SbF_5, −60 °C.
Figure S15. Stacked ^{19}F NMR (top) and ^{13}C NMR (bottom) spectra of $C_2O_2F_2$ and $[C_2O(OH)Cl_2][SbF_6]$ (**1**) in aHF, −60 °C.
Figure S16. ^1H and ^{19}F NMR spectra of $C_2O_2Cl_2$ in HF/8 SbF_5, −60 °C.
Figure S17. ^{13}C NMR spectrum of $C_2O_2Cl_2$ in HF/8 SbF_5, −60 °C.
Figure S18. Stacked ^{19}F NMR spectra of $C_2O_2F_2$ (top) and COF_2 (bottom) against $[C_2(OH)_2Cl_2][Sb_nF_{5n+1}]$ (**3**) in aHF, −60 °C.

Tables:

Table S1. Low-temperature Raman and IR spectra of $[C_2O(OH)Cl_2][SbF_6]$ (**1**) and $[C_2O(OD)Cl_2][SbF_6]$ (**2**) and calculated vibrational frequencies [cm^{-1}] of $[C_2O(OH)Cl_2]^+\cdot HF$ (C_S symmetry).
Table S2. Low-temperature Raman spectrum of $[ClCO][Sb_3F_{15}Cl]$ (**4**) and calculated vibrational frequencies [cm^{-1}] of $[ClCO]^+\cdot HF$ ($C_{\infty v}$ symmetry).[11]
Table S3. Low-temperature Raman spectrum of $[ClCO][Sb_3F_{16}]$ (**5**) and calculated vibrational frequencies [cm^{-1}] of $[ClCO]^+\cdot HF$ ($C_{\infty v}$ symmetry).[11]
Table S4. Low-temperature Raman and IR spectra of $C_2O_2Cl_2$ and calculated vibrational frequencies [cm^{-1}] of $C_2O_2Cl_2$ (C_{2h} symmetry).[12]
Table S5. Low-temperature Raman and IR spectra of ClFCO and calculated vibrational frequencies [cm^{-1}] of ClFCO (C_S symmetry).[13]
Table S6. X-ray data and refinement of $[C_2O(OH)Cl_2][SbF_6]$ (**1**) and $[ClCO][Sb_3F_{16}]$ (**5**).
Table S7. Bond lengths [Å] and bond angles [°] of $[C_2O(OH)Cl_2][SbF_6]$ (**1**) as well as donor-acceptor distances of **1**.
Table S8. Bond lengths [Å] and bond angles [°] of $[ClCO][Sb_3F_{16}]$ (**5**) as well as donor-acceptor distances of **5**.
Table S9. Standard orientations of $[C_2O(OH)Cl_2]^+\cdot HF$. Calculated at the ωB97XD/aug-cc-pVTZ-level of theory.
Table S10. Standard orientations of $[ClCO]^+\cdot HF$. Calculated at the ωB97XD/aug-cc-pVTZ-level of theory.
Table S11. Standard orientations of $C_2O_2Cl_2$. Calculated at the ωB97XD/aug-cc-pVTZ-level of theory.
Table S12. Standard orientations of ClFCO. Calculated at the ωB97XD/aug-cc-pVTZ-level of theory.

Experimental Section

Caution! Avoid skin contact with all compounds. Hydrolysis can lead to the formation of HF or DF that burns skin and causes irreparable damage. Ensure appropriate safety precautions while handling these materials.

Apparatus and Materials: All reactions were performed employing standard Schlenk techniques using a stainless-steel vacuum line. Syntheses were carried out using FEP/PFA tube reactors closed with stainless-steel valves. Before use, the stainless-steel vacuum line and all reactors were dried with fluorine. Low-temperature Raman spectra were recorded under vacuum using glass cells cooled with liquid nitrogen and a Bruker® MultiRAMII FT Raman spectrometer equipped with Nd:YAG laser (λ = 1064 nm) and a laser excitation of 500−1000 mW. Low-temperature IR spectra were recorded with a Bruker® Vertex FT IR spectrometer at −196 °C. For measurements, low-temperature IR cells[1] were prepared with CsBr single-crystal plates coated with a small amount of the samples. For visualization, the software Advanced Chemistry Development Inc.® (ACD/Labs 2015) was employed. Low-temperature X-ray diffraction was performed with an Oxford XCalibur3 diffractometer equipped with a Spellman Generator (50 kV, 40 mA) and a Kappa CCD detector, using Mo-Kα radiation (λ = 0.71073 Å). The program CrysAlisPro 1.171.40.84a (Rigaku OD, 2020)[2] was employed for the data collection and reduction. The structure solution and refinement were performed with the software SHELXT[3] and SHELXL-2019/3,[4] implemented in the WinGX software package.[5] The solution was checked with the program PLATON[6] and the absorption correction was performed using the SCALE3 ABSPACK multi-scan-method.[7] Selected data and parameters of the single-crystal X-ray structure analyses are summarized in Table S6 for **1** and **5**, respectively (see Supporting Information). Crystallographic data (excluding structure factors) for the structures in this paper were deposited at the Cambridge Crystallographic Data Centre, CCDC, 12 Union Road, Cambridge CB21EZ, UK. Copies of the data can be obtained free of charge by quoting the depository number CCDC-2304777 for $[C_2O(OH)Cl_2][SbF_6]$ (**1**) and CCDC-2304793 for $[ClCO][Sb_3F_{16}]$ (**5**) (Fax: +44-1223-336-033; E-Mail: deposit@ccdc.cam.ac.uk, http://www.ccdc.cam.ac.uk). NMR samples were prepared by adding the HF solution to a small FEP tube under a nitrogen stream. The tube was sealed under a vacuum and inserted into a standard NMR tube. For ^{1}H, ^{19}F, and ^{13}C NMR measurements a JEOL ECX 400 NMR spectrometer was used. For evaluation, MNOVA by Mestrelab was used.[8] Quantum chemical calculations were performed at the ωB97XD/aug-cc-pVTZ-level of theory by Gaussian16.[9] GaussView 6.0 was used for visualization and illustration of the MEP calculations.[10]

Synthesis of $[C_2O(OH)Cl_2][SbF_6]$: Antimony pentafluoride (1 mmol, 217 mg, 1.0 eq.) and anhydrous hydrogen fluoride (100 mmol, 2 mL) were condensed into an FEP tube-reactor at −196 °C. To form the superacid, the mixture was warmed up to −50 °C and both components were mixed. After the mixture was cooled to −196 °C again, oxalyl chloride (1 mmol, 127 mg, 1.0 eq.) was condensed into the reaction vessel. Subsequently, the mixture was warmed to −60 °C and all components were reacted. Then, the temperature was reduced to −78 °C. The remaining HF was removed in a dynamic vacuum at −78 °C. Compound (**1**) was obtained in quantitative yield as a colorless solid. Compound (**2**) was prepared analogously, using *a*DF instead of *a*HF.

Synthesis of $[C_2(OH)_2Cl_2][Sb_nF_{5n+1}]$ (3): Antimony pentafluoride (3.28 mmol, 710 mg, 8.0 eq.) and anhydrous hydrogen fluoride (100 mmol, 2 mL) were condensed into an FEP tube-reactor at −196 °C. To form the superacid, the mixture was warmed up to −50 °C and both components were mixed. After the mixture was cooled to −196 °C again, oxalyl chloride (0.409 mmol, 52 mg, 1.0 eq.) was condensed into the reaction vessel. Subsequently, the mixture was warmed to −60 °C and all components were reacted. For NMR measurements, the samples were prepared as mentioned before.

Synthesis of $[ClCO][Sb_3F_{15}Cl]$: Antimony pentafluoride (1.5 mmol, 326 mg, 3.0 eq.) and 1,1,1,2-tetrafluoroethane (R-134a) (50 mmol, 4.2 mL) were condensed into an FEP tube-reactor at −196 °C. Then, the mixture was warmed up to −30 °C and both components were mixed. After the mixture was cooled to −196 °C again, oxalyl chloride (0.5 mmol, 63 mg, 1.0 eq.) was condensed into the reaction vessel. Subsequently, the mixture was warmed to −78 °C and all components were reacted. Then, the temperature was reduced to −78 °C. The remaining R-134a was removed in a dynamic vacuum at −78 °C. Compound (**4**) was obtained in quantitative yield as a colorless solid. $[ClCO][Sb_3F_{16}]$ (**5**) was prepared analogously, using carbonyl chloride fluoride (0.5 mmol, 41 mg, 1.0 eq.) instead of oxalyl chloride.

Table S1: Low-temperature Raman and IR spectra of $[C_2O(OH)Cl_2][SbF_6]$ (**1**) and $[C_2O(OD)Cl_2][SbF_6]$ (**2**) and calculated vibrational frequencies [cm^{-1}] of $[C_2O(OH)Cl_2]^{+}\cdot HF$ (C_S symmetry).

$[C_2O(OH)Cl_2][SbF_6]$ (**1**)		$[C_2O(OD)Cl_2][SbF_6]$ (**2**)		$[C_2O(OH)Cl_2]^{+}\cdot HF$	Assignment		
Raman	IR	Raman	IR	calc.[a,b] (IR/Raman)[c]			
			3508 w				
			3157 w				H_2O
				2932(1978/128)	ν_1	A'	ν_s(O–H)
			2849 w				
	2484 m						
			2388 vs				
		2286(7)					
		2157(4)					ν_s(O–D)
	2150 vw						
			1857 w				$C_2O_2Cl_2$
1779(16)	1774 m	1772(9)	1772 m	1799(206/18)	ν_2	A'	ν_s(C=O)
		1764(21)					$C_2O_2Cl_2$
			1726 w				
1607(5)	1605 w	1607(5)	1593 vw	1575(179/8)	ν_3	A'	ν_s(C=O)
			1468 w				
1329(2)	1319 w		1313 w	1326(249/5)	ν_4	A'	δ_s(COH)
			1265 w				
			1229 w				
		1195(3)	1194 w				
			1148 w				
			1138 w				
1118(4)	1117 w		1117 w	1056(23/11)	ν_5	A'	ν_s(C–C)
1095(3)		1095(4)					$C_2O_2Cl_2$
	1045 m						
		1025(5)					
		993(12)					
		982(15)	986 w				
	962 w		966 w				
	924 m		922 w	939(91/0)	ν_{12}	A''	κ_{as}(COH)
		890(3)					
	851 w						
	833 w	817(12)	818 m	832(310/1)	ν_6	A'	ν_s(C–Cl)
			754 m				
712(7)		710(5)		707(0/0)	ν_{13}	A''	κ_{as}(CCO)
		623(30)		610(6/17)	ν_7	A'	ν_s(C–Cl)

525(4)	525 vw	526(6)		520(11/0)	ν_8	A'	δ_s(COCl)
466(13)	476 m	445(9)	446 s	456(57/4)	ν_9	A'	δ_s(COCl)
		429(13)					$C_2O_2Cl_2$
	399 m		382 m	383(5/0)	ν_{14}	A''	κ_{as}(COCl)
238(5)				245(11/1)	ν_{10}	A'	δ_s(ClCCO)
182(4)		167(7)		165(5/0)	ν_{11}	A'	δ_s(ClCCO)
				12(0/0)	ν_{15}	A''	τ_{as}(CCCl)
681(21)	685 s	690(13)					ν(Sb–F)
668(100)	669 s	672(35)					ν(Sb–F)
		658(100)	656 m				ν(Sb–F)
639(54)	636 w	639(26)					ν(Sb–F)
589(17)	588 w	591(13)					ν(Sb–F)
560(4)		553(8)					ν(Sb–F)
		491(8)	486 w				δ(Sb–F)
		399(14)					δ(Sb–F)
		391(14)	390 m				δ(Sb–F)
	374 w						δ(Sb–F)
	361 w		359 m				δ(Sb–F)
294(21)							δ(Sb–F)
284(18)		287(31)					δ(Sb–F)
268(19)		268(14)					δ(Sb–F)
230(5)		230(7)					δ(Sb–F)
193(4)							δ(Sb–F)
136(14)							δ(Sb–F)
117(10)							δ(Sb–F)

[a] Calculated at ωB97XD/aug-cc-pVTZ-level of theory, [b] Frequencies are scaled with a factor of 0.956, [c] IR intensity in [km/mol] and Raman intensity in [Å^4/u]. Abbreviations for IR intensities: vs = very strong, s = strong, m = medium, w = weak.

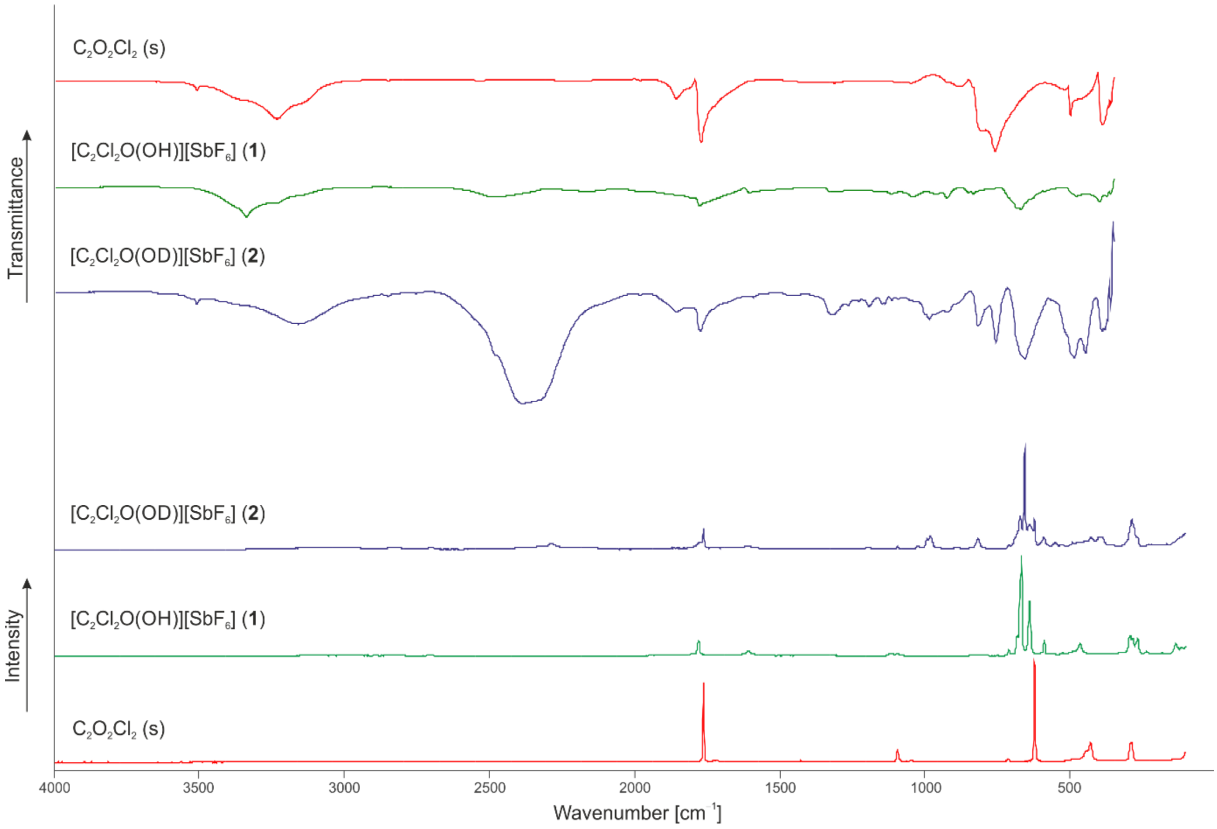

Figure S1: Low-temperature Raman and IR spectra of $[C_2O(OH)Cl_2][SbF_6]$ (**1**), $[C_2O(OD)Cl_2][SbF_6]$ (**2**) and $C_2O_2Cl_2$.

Table S2: Low-temperature Raman spectrum of $[ClCO][Sb_3F_{15}Cl]$ (**4**) and calculated vibrational frequencies [cm^{-1}] of $[ClCO]^+\cdot HF$ ($C_{\infty v}$ symmetry).[11]

$[ClCO][Sb_3F_{15}Cl]$ (**4**) Raman	$[ClCO]^+\cdot HF$ calc.[a,b] (IR/Raman)[c]	Assignment		
2256(31)	2256(366/33)	ν_1	Σ	ν(C≡O)
947(3)				
841(5)				
804(22)				ν(C–Cl)
795(10)	784(13/12)	ν_2	Σ	ν(C–Cl)
463(16)	489(16/0)	ν_3	π	δ(ClCO)
706(56)				ν(Sb–F)
696(28)				ν(Sb–F)
685(50)				ν(Sb–F)
673(24)				ν(Sb–F)
655(100)				ν(Sb–F)
613(9)				ν(Sb–F)
605(17)				ν(Sb–F)
453(19)				δ(Sb–F)
396(52)				δ(Sb–Cl)
324(9)				δ(Sb–F)
302(25)				δ(Sb–F)
278(19)				δ(Sb–F)
254(8)				δ(Sb–F)
239(17)				δ(Sb–F)
225(20)				δ(Sb–F)
214(12)				δ(Sb–F)
193(13)				δ(Sb–F)
134(28)				δ(Sb–F)
114(24)				δ(Sb–F)

[a] Calculated at ωB97XD/aug-cc-pVTZ-level of theory, [b] Frequencies are scaled with a factor of 0.956, [c] IR intensity in [km/mol] and Raman intensity in [Å^4/u]. Abbreviations for IR intensities: vs = very strong, s = strong, m = medium, w = weak.

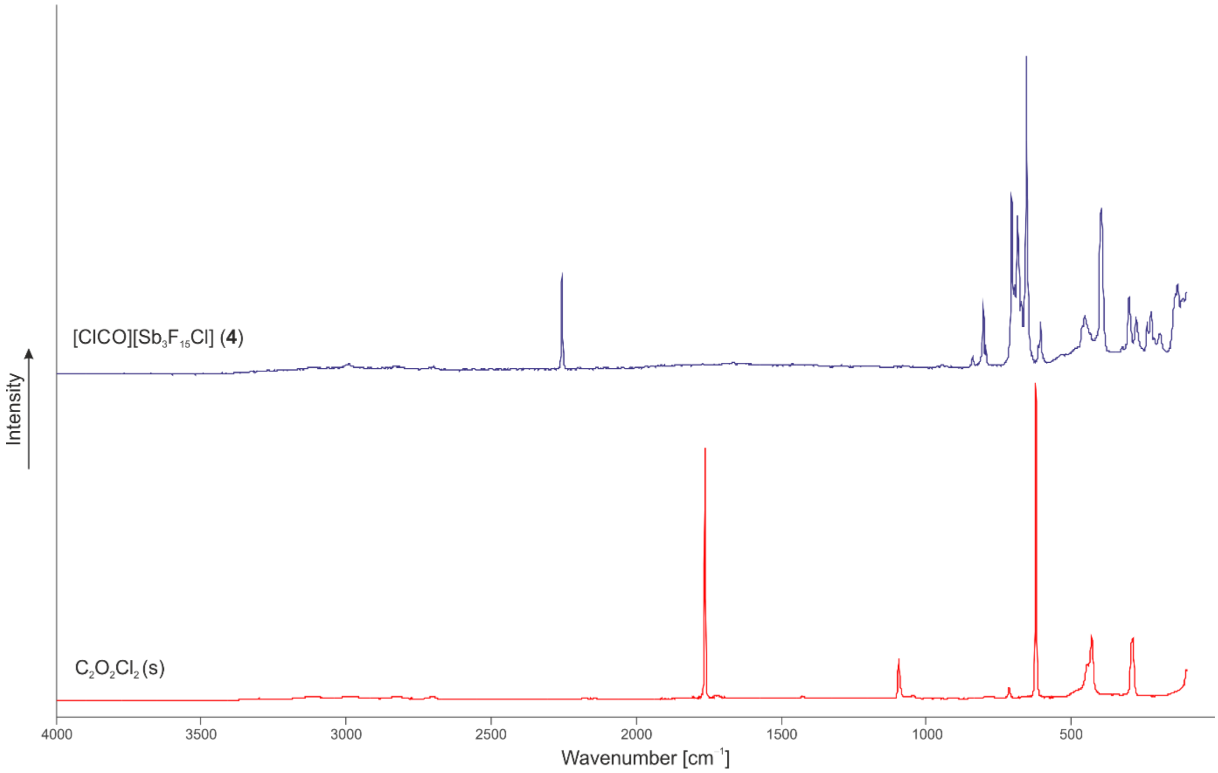

Figure S2: Low-temperature Raman spectra of [ClCO][$Sb_3F_{15}Cl$] (**4**) and $C_2O_2Cl_2$.

Table S3: Low-temperature Raman spectrum of $[ClCO][Sb_3F_{16}]$ (**5**) and calculated vibrational frequencies [cm^{-1}] of $[ClCO]^{+}\cdot HF$ ($C_{\infty v}$ symmetry).[11]

$[ClCO][Sb_3F_{16}]$ (**5**) Raman	$[ClCO]^{+}\cdot HF$ calc.[a,b] (IR/Raman)[c]	Assignment		
2256(20)	2256(366/33)	ν_1	Σ	ν(C≡O)
947(4)				$2\nu_3$
841(6)				
804(17)				ν(C–Cl)
795(9)	784(13/12)	ν_2	Σ	ν(C–Cl)
465(8)	489(16/0)	ν_3	π	δ(ClCO)
749(8)				ν(Sb–F)
717(100)				ν(Sb–F)
706(47)				ν(Sb–F)
685(34)				ν(Sb–F)
671(97)				ν(Sb–F)
655(72)				ν(Sb–F)
613(9)				ν(Sb–F)
606(12)				ν(Sb–F)
321(11)				δ(Sb–F)
303(20)				δ(Sb–F)
278(36)				δ(Sb–F)
239(19)				δ(Sb–F)
229(14)				δ(Sb–F)
205(12)				δ(Sb–F)
188(11)				δ(Sb–F)
137(19)				δ(Sb–F)

[a] Calculated at ωB97XD/aug-cc-pVTZ-level of theory, [b] Frequencies are scaled with a factor of 0.956, [c] IR intensity in [km/mol] and Raman intensity in [Å^4/u]. Abbreviations for IR intensities: vs = very strong, s = strong, m = medium, w = weak.

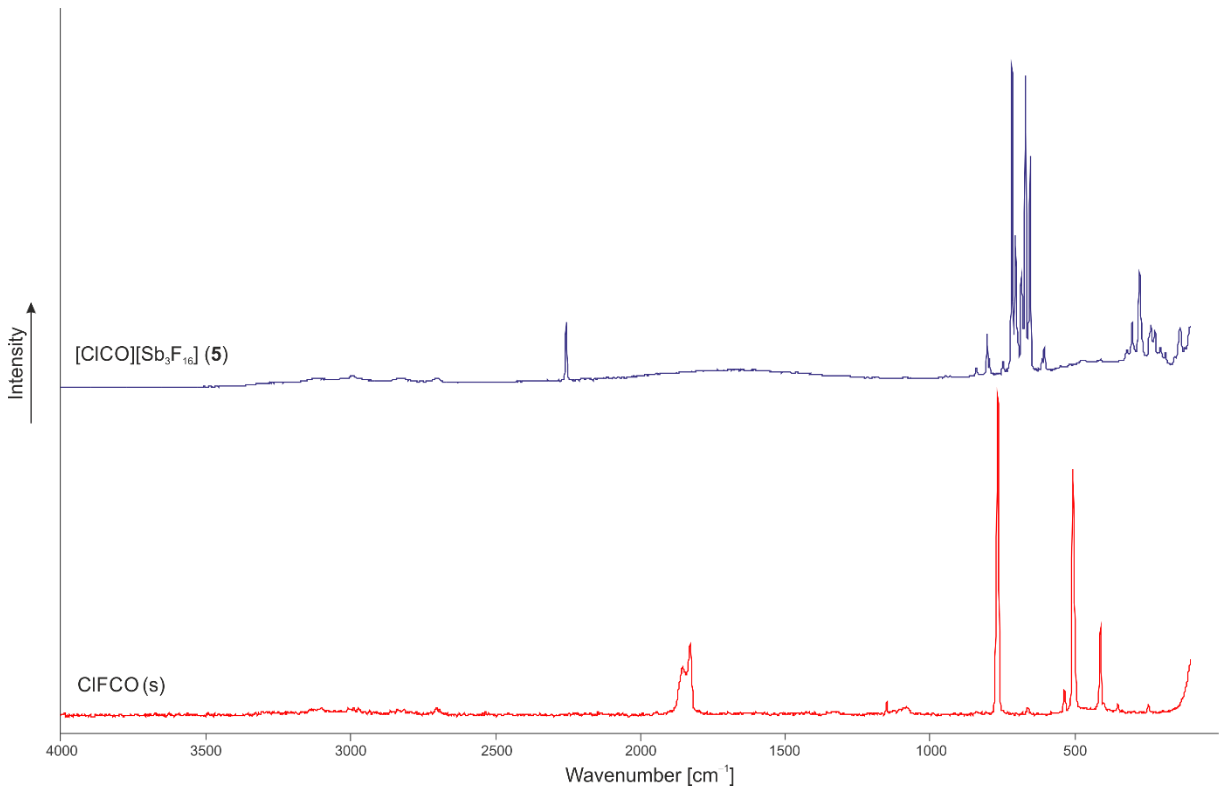

Figure S3: Low-temperature Raman spectra of $[ClCO][Sb_3F_{16}]$ (**5**) and ClFCO.

Table S4: Low-temperature Raman and IR spectra of $C_2O_2Cl_2$ and calculated vibrational frequencies [cm^{-1}] of $C_2O_2Cl_2$ (C_{2h} symmetry).[12]

$C_2O_2Cl_2$		$C_2O_2Cl_2$	Assignment		
Raman	IR	calc.[a,b] (IR/Raman)[c]			
	1857 w				
	1770 s	1829(467/0)	ν_9	B_u	ν(C=O)
1764(79)		1802(0/43)	ν_1	A_g	ν(C=O)
1095(13)	1099 w	1030(0/7)	ν_2	A_g	ν(C–C)
1047(2)	1049 w				
	876 w				
	806 m				
	756 s	743(534/0)	ν_{10}	B_u	ν(C–Cl)
713(4)		706(0/0)	ν_6	B_g	κ(COCl)
623(100)		591(0/28)	ν_3	A_g	ν(C–Cl)
	518 w				
	498 m	478(7/0)	ν_{11}	B_u	δ(COCl)
444(12)					
430(20)		416(0/10)	ν_4	A_g	δ(COCl)
	390 m	376(17/0)	ν_7	A_u	κ(COCl)
292(19)					
287(20)		274(0/6)	ν_5	A_g	δ(CCCl)
103(10)		195(4/0)	ν_{12}	B_u	δ(CCCl)
		15(1/0)	ν_8	A_u	τ(CCCl)

[a] Calculated at ωB97XD/aug-cc-pVTZ-level of theory, [b] Frequencies are scaled with a factor of 0.956, [c] IR intensity in [km/mol] and Raman intensity in [$Å^4$/u]. Abbreviations for IR intensities: vs = very strong, s = strong, m = medium, w = weak.

Table S5: Low-temperature Raman and IR spectra of ClFCO and calculated vibrational frequencies [cm^{-1}] of ClFCO (C_S symmetry).[13]

ClFCO		ClFCO	Assignment		
Raman	IR	calc.[a,b] (IR/Raman)[c]			
	2648 w				ν_1+ν_3
	2357 w				
	2187 m				
	2172 m				2ν_2
1854(16)	1867 vs	1864(430/14)	ν_1	A'	ν_s(C=O)
1829(22)					ν_2+ν_3
	1340 w				
	1325 w				2ν_6
	1271 m				
	1256 m				
1150(5)	1178 w				ν_4+ν_6
1083(3)	1103 vs	1076(424/2)	ν_2	A'	ν_s(C–F)
	1009 w				2ν_4
	989 w				
	910 w				ν_4+ν_5
	847 s				2ν_5
767(100)	771 vs	742(94/10)	ν_3	A'	ν_s(C–Cl)+ δ(COF)
	756 vs				
	683 s				
663(3)	671 s	655(15/0)	ν_6	A''	κ(COF)
537(9)					
507(78)	513 w	482(1/6)	ν_4	A'	δ(COCl)
413(28)	395 m	395(0/1)	ν_5	A'	δ(ClCF)
401(5)					
352(4)					
248(4)					

[a] Calculated at ωB97XD/aug-cc-pVTZ-level of theory, [b] Frequencies are scaled with a factor of 0.956, [c] IR intensity in [km/mol] and Raman intensity in [Å^4/u]. Abbreviations for IR intensities: vs = very strong, s = strong, m = medium, w = weak.

Table S6: X-ray data and refinement of $[C_2O(OH)Cl_2][SbF_6]$ (**1**) and $[ClCO][Sb_3F_{16}]$ (**5**).

	$[C_2O(OH)Cl_2][SbF_6]$ (**1**)	$[ClCO][Sb_3F_{16}]$ (**5**)
formula	$C_2HCl_2F_6O_2Sb$	$CClF_{16}OSb_3$
M_r [g mol^{-1}]	363.68	732.71
crystal size [mm^3]	0.306 x 0.171 x 0.118	0.190 x 0.136 x 0.106
crystal system	monoclinic	trigonal
space group	$P2_1$	$P3_1$
a [Å]	6.1616(8)	8.0824(3)
b [Å]	10.8379(10)	8.0824(3)
c [Å]	6.8805(8)	18.3341(8)
α [°]	90	90
β [°]	106.472(13)	90
γ [°]	90	120
V [Å³]	440.61(9)	1037.22(9)
Z	2	3
ρ_{calc} [g cm^{-3}]	2.741	3.519
μ [mm^{-1}]	3.801	6.191
$\lambda_{MoK\alpha}$ [Å]	0.71073	0.71073
F(000)	336	984
T [K]	106(2)	102(2)
hkl range	–9:9, –15:15, –10:10	–9:12, –12:9, –23:27
refl. measured	8919	6310
refl. unique	2930	3334
R_{int}	0.0415	0.0436
parameters	122	199
R(F)/wR(F²) [a] (all reflexions)	0.0364/0.0570	0.0462/0.0767
weighting scheme [b]	0.0193/0.0000	0.0262/0.0000
S (GooF) [c]	1.042	1.032
residual density [e Å^{-3}]	1.183/–0.655	1.356/–1.093
device type	Oxford XCalibur	Oxford XCalibur
solution	SHELXT[3]	SHELXT[3]
refinement	SHELXL-2019/3[4]	SHELXL-2019/3[4]
CCDC	2304777	2304793

[a] $R_1 = \Sigma\,||F_0|-|F_c||/\Sigma|F_0|$;
[b] $wR_2 = [\Sigma[w(F^2_0 - F^2_C)^2]/\Sigma(F_0)^2]]^{1/2}$; $w = [\sigma^2_C(F^2_0) + (xP)^2 + yP]^{-1}$; $P = (F^2_0 + 2F^2_C)/3$;
[c] $GooF = [\Sigma[(F^2_0 - F^2_C)^2]/(n-p)]^{1/2}$ (*n* = number of reflexions; *p* = total numbers of parameters).

Details on crystal structure refinement:

$[C_2O(OH)Cl_2][SbF_6]$ (**1**)

The structure was refined as an inversion twin due to a flack parameter close to 0.5. Using the difmap, the contact of 2.421 Å (strong H bond) between O1···F6 and the enlarged CO bond distance revealed the protonation of the acyl halide function. As no peak of rest electron density could be identified, the position of the proton was modulated with AFIX147 command and refined under restriction of a DFIX command.

$[ClCO][Sb_3F_{16}]$ (**5**)

The structure was refined as a twin with 4 domains according to the proposed TWIN law (1-2 0) reported by Checkcif. As visualized by the BASF, one of the four domains was fully cut off. The R values as well as the electronic structure of the carbon ellipsoid justify this refinement as a better solution compared with the refinement as inversion twin. Therefore DFIX, as well as OMIT commands are not needed anymore. As the target resolution of the measurement was set to 0.7 Å, a SHEL1000 command was set to cut of weak diffracting areas in accordance with the K value.

Table S7: Bond lengths [Å] and bond angles [°] of $[C_2O(OH)Cl_2][SbF_6]$ (**1**) as well as donor-acceptor distances of **1**.

$[C_2O(OH)Cl_2][SbF_6]$ (**1**)			
Bond lengths [Å]			
C1–Cl1	1.647(7)	Sb1–F2	1.863(4)
C1–O1	1.225(8)	Sb1–F3	1.848(4)
C1–C2	1.55(1)	Sb1–F4	1.856(3)
C2–Cl2	1.693(7)	Sb1–F5	1.847(4)
C2–O2	1.185(8)	Sb1–F6	1.942(3)
Sb1–F1	1.845(4)		
Bond angles [°]			
Cl1–C1–O1	124.9(5)	F2–Sb1–F3	91.6(2)
Cl1–C1–C2	117.2(5)	F2–Sb1–F4	88.5(2)
O1–C1–C2	117.9(6)	F2–Sb1–F5	174.0(2)
C1–C2–Cl2	114.0(5)	F2–Sb1–F6	85.9(2)
O2–C2–C1	119.4(6)	F3–Sb1–F4	92.5 (2)
O2–C2–Cl2	126.6(6)	F3–Sb1–F5	94.5(2)
F1–Sb1–F2	89.3(2)	F3–Sb1–F6	177.3(2)
F1–Sb1–F3	92.5(2)	F4–Sb1–F5	91.1(2)
F1–Sb1–F4	174.6(2)	F4–Sb1–F6	86.6(2)
F1–Sb1–F5	90.6(2)	F5–Sb1–F6	88.1(2)
F1–Sb1–F6	88.3(2)		
Dihedral angles [°]			
Cl1–C1–C2–Cl2	−177.4(4)	O1–C1–C2–O2	−175.1(7)
Cl1–C1–C2–O2	4.9(9)	O1–C1–C2–Cl2	2.6(8)
Donor-acceptor distances [Å]			
O1(–H2)···F6	2.421(7)	C2···F4*ii*	2.809(7)
C1···F2*i*	2.617(7)	Cl1···F3*iii*	2.883(5)
C1···F4*ii*	2.565(7)	F1···F4*iii*	2.832(6)
C2···F2*i*	3.026(8)	O1···F2*i*	2.843(6)

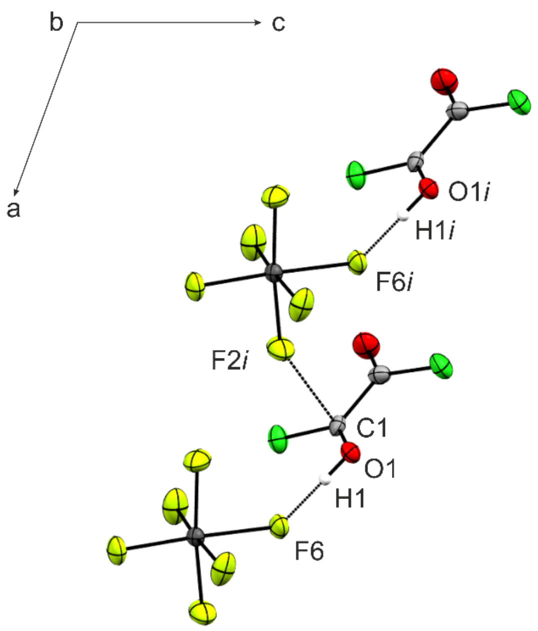

Figure S4: Chains along the *a*-axis of **1** (displacement ellipsoids with 50% probability).
Symmetry codes: *i* = –1+*x*, *y*, *z*; *ii* = 1–*x*, 0.5+*y*, 1–*z*; *iii* = 1–*x*, 0.5+*y*, –*z*.

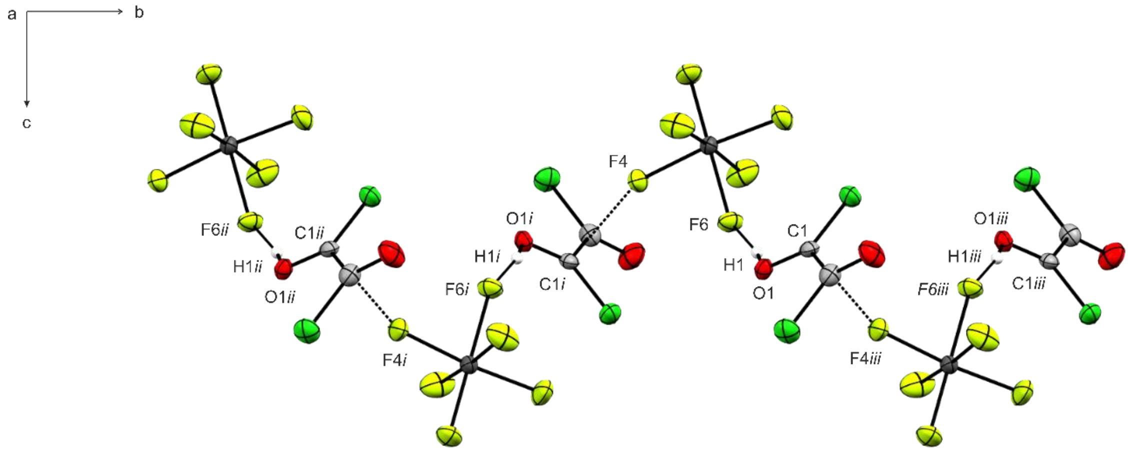

Figure S5: Chains along the *b*-axis of **1** (displacement ellipsoids with 50% probability).
Symmetry codes: *i* = –1+*x*, *y*, *z*; *ii* = 1–*x*, 0.5+*y*, 1–*z*; *iii* = 1–*x*, 0.5+*y*, –*z*.

Table S8: Bond lengths [Å] and bond angles [°] of [ClCO][Sb_3F_{16}] (**5**) as well as donor-acceptor distances of **5**.

[ClCO][Sb_3F_{16}] (**5**)			
Bond lengths [Å]			
C1–O1	1.105(10)	Sb2–F8	1.830(6)
C1–Cl1	1.571(3)	Sb2–F9	1.84(2)
Sb1–F1	1.85(1)	Sb2–F10	1.83(1)
Sb1–F2	1.83(1)	Sb2–F11	1.98(2)
Sb1–F3	1.83(2)	Sb3–F11	2.09(1)
Sb1–F4	1.84(1)	Sb3–F12	1.857(8)
Sb1–F5	1.85(1)	Sb3–F13	1.85(1)
Sb1–F6	2.07(1)	Sb3–F14	1.855(7)
Sb2–F6	1.99(1)	Sb3–F15	1.84(2)
Sb2–F7	1.835(6)	Sb3–F16	1.855(9)
Bond angles [°]			
Cl1–C1–O1	176.1(8)	F7–Sb2–F10	94.4(5)
F1–Sb1–F2	94.3(5)	F7–Sb2–F11	84.7(5)
F1–Sb1–F3	95.5(5)	F8–Sb2–F9	94.5(5)
F1–Sb1–F4	95.3(5)	F8–Sb2–F10	93.8(5)
F1–Sb1–F5	94.6(6)	F8–Sb2–F11	83.8(5)
F1–Sb1–F6	178.6(5)	F9–Sb2–F10	98.3(5)
F2–Sb1–F3	89.0(5)	F9–Sb2–F11	172.8(5)
F2–Sb1–F4	91.9(5)	F10–Sb2–F11	88.8(5)
F2–Sb1–F5	170.8(5)	F11–Sb3–F12	83.0(5)
F2–Sb1–F6	84.6(4)	F11–Sb3–F13	84.4(5)
F3–Sb1–F4	169.1(5)	F11–Sb3–F14	85.0(4)
F3–Sb1–F5	88.0(5)	F11–Sb3–F15	179.1(5)
F3–Sb1–F6	85.3(4)	F11–Sb3–F16	83.6(4)
F4–Sb1–F5	89.3(5)	F12–Sb3–F13	89.3(5)
F4–Sb1–F6	83.9(4)	F12–Sb3–F14	168.0(5)
F5–Sb1–F6	86.5(4)	F12–Sb3–F15	96.2(5)
F6–Sb2–F7	85.3(5)	F12–Sb3–F16	89.7(5)
F6–Sb2–F8	85.0(5)	F13–Sb3–F14	90.0(5)
F6–Sb2–F9	89.4(5)	F13–Sb3–F15	95.1(5)
F6–Sb2–F10	172.3(5)	F13–Sb3–F16	168.0(5)
F6–Sb2–F11	83.5(4)	F14–Sb3–F15	95.8(5)
F7–Sb2–F8	165.7(5)	F14–Sb3–F16	88.4(5)
F7–Sb2–F9	95.9(5)	F15–Sb3–F16	98.9(5)
Sb1–F6–Sb2	145.9(5)	Sb2–F11–Sb3	156.7(6)
Dihedral angles [°]			

F1–Sb1–F6–Sb2	2(21)	F16–Sb3–F11–Sb2	50(1)
F9-Sb2–F11–Sb3	173(3)	F10–Sb2–F11–Sb3	–16(2)
Donor-acceptor distances [Å]			
C1···F2*i*	3.063	Cl1···F1	2.56(1)
C1···F4*ii*	2.968	O1···F2*i*	2.91(1)
C1···F5*iii*	2.75	O1···F3*iii*	2.92(2)
C1···F14*i*	3.062	O1···F10*v*	2.81(2)
C1···F15*iv*	2.92	O1···F16*v*	2.79(2)

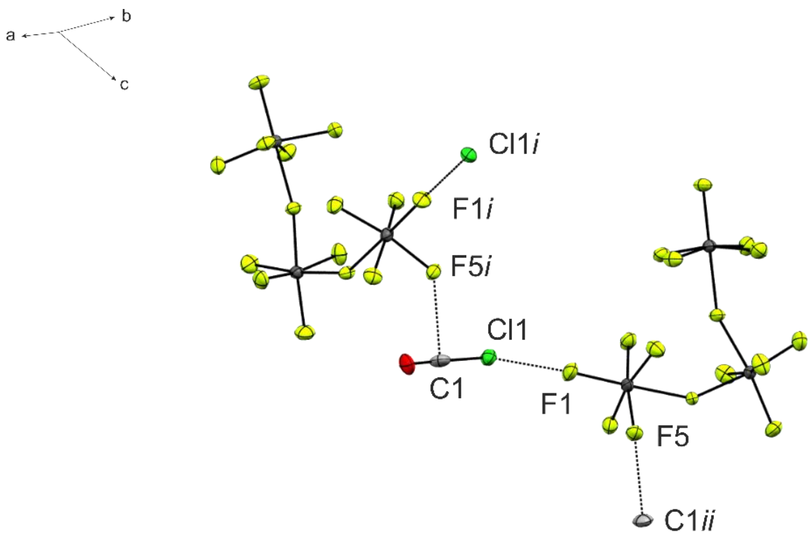

Figure S6: Chains along the *c*-axis of **5** (displacement ellipsoids with 50% probability). Symmetry codes: *i* = –*x*+*y*, 1–*x*, –1/3+*z*; *ii* = 1–*y*, 1+*x*–*y*, 1/3+*z*.

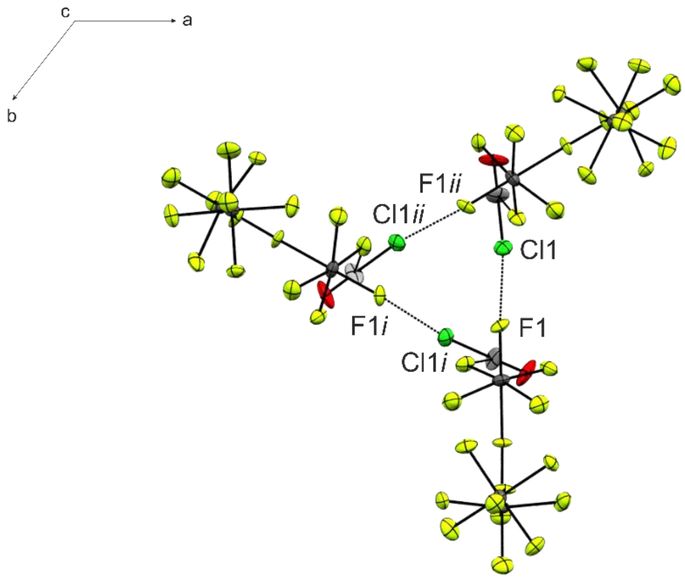

Figure S7: Helical structure along the *c*-axis of **5** (displacement ellipsoids with 50% probability). Symmetry codes: *i* = –*y*, *x*–*y*, –2/3+*z*; *ii* = –*x*+*y*, –*x*, –1/3+*z*.

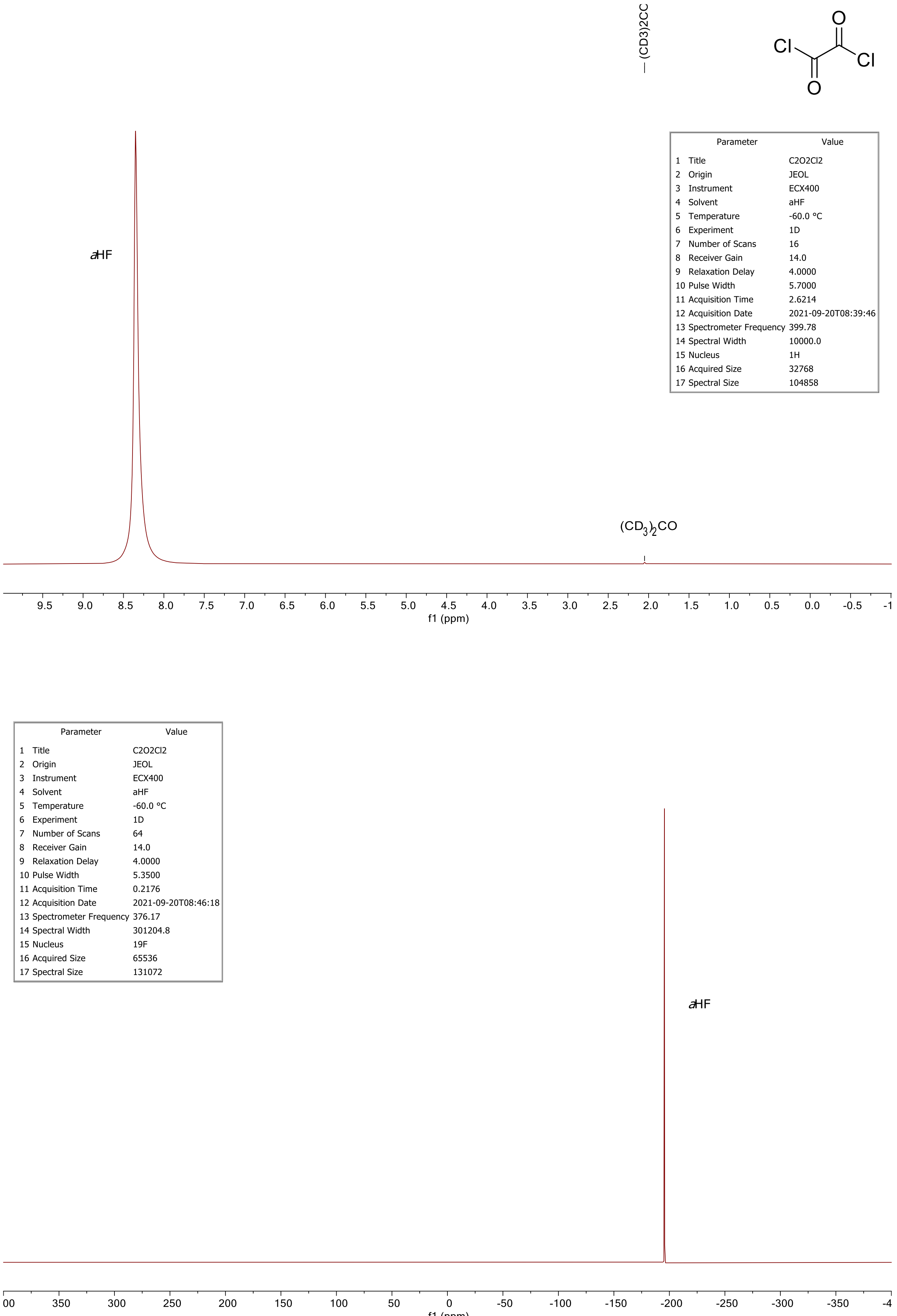

	Parameter	Value
1	Title	C2O2Cl2
2	Origin	JEOL
3	Instrument	ECX400
4	Solvent	aHF
5	Temperature	-60.0 °C
6	Experiment	1D
7	Number of Scans	16
8	Receiver Gain	14.0
9	Relaxation Delay	4.0000
10	Pulse Width	5.7000
11	Acquisition Time	2.6214
12	Acquisition Date	2021-09-20T08:39:46
13	Spectrometer Frequency	399.78
14	Spectral Width	10000.0
15	Nucleus	1H
16	Acquired Size	32768
17	Spectral Size	104858

	Parameter	Value
1	Title	C2O2Cl2
2	Origin	JEOL
3	Instrument	ECX400
4	Solvent	aHF
5	Temperature	-60.0 °C
6	Experiment	1D
7	Number of Scans	64
8	Receiver Gain	14.0
9	Relaxation Delay	4.0000
10	Pulse Width	5.3500
11	Acquisition Time	0.2176
12	Acquisition Date	2021-09-20T08:46:18
13	Spectrometer Frequency	376.17
14	Spectral Width	301204.8
15	Nucleus	19F
16	Acquired Size	65536
17	Spectral Size	131072

Figure S8: ^{1}H and ^{19}F NMR spectra of $C_2O_2Cl_2$ in *a*HF, –60 °C.

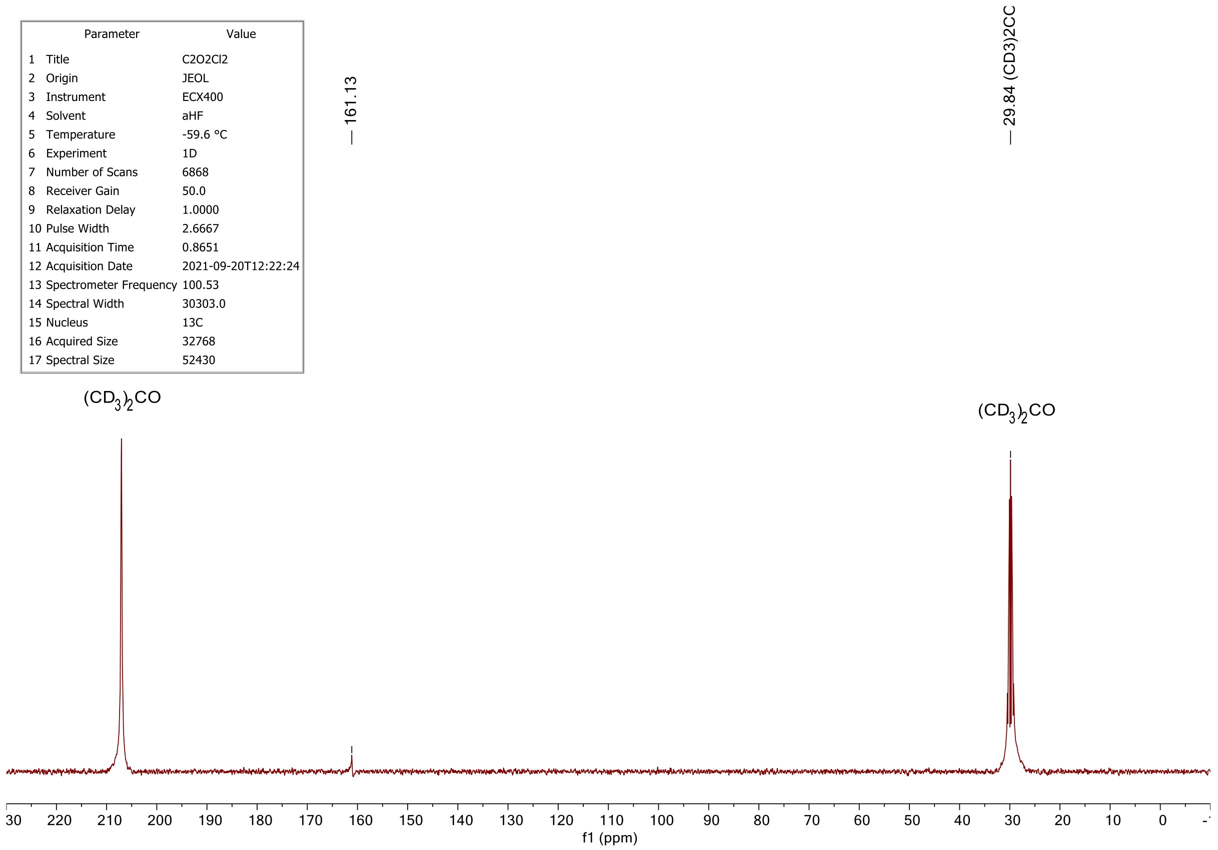

	Parameter	Value
1	Title	C2O2Cl2
2	Origin	JEOL
3	Instrument	ECX400
4	Solvent	aHF
5	Temperature	-59.6 °C
6	Experiment	1D
7	Number of Scans	6868
8	Receiver Gain	50.0
9	Relaxation Delay	1.0000
10	Pulse Width	2.6667
11	Acquisition Time	0.8651
12	Acquisition Date	2021-09-20T12:22:24
13	Spectrometer Frequency	100.53
14	Spectral Width	30303.0
15	Nucleus	13C
16	Acquired Size	32768
17	Spectral Size	52430

Figure S9: ^{13}C NMR spectrum of $C_2O_2Cl_2$ in *a*HF, –60 °C.

$C_2O_2Cl_2$:

^{13}C NMR [101 MHz, –60 °C, $(CD_3)_2CO$)]: δ = 161.1 (s, CO).

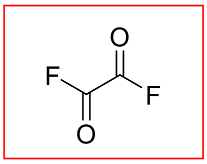

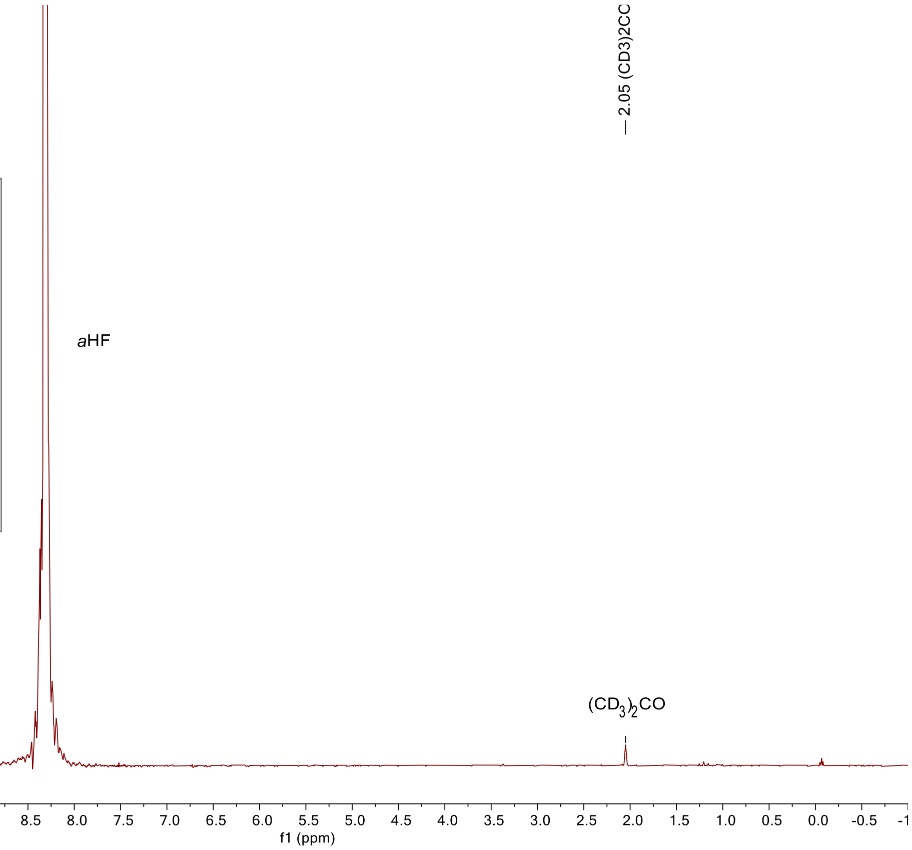

Parameter	Value
1 Title	C2O2F2
2 Origin	JEOL
3 Instrument	ECX400
4 Solvent	aHF
5 Temperature	-60.0 °C
6 Experiment	1D
7 Number of Scans	16
8 Receiver Gain	30.0
9 Relaxation Delay	4.0000
10 Pulse Width	5.7000
11 Acquisition Time	2.6214
12 Acquisition Date	2021-03-12T08:47:37
13 Spectrometer Frequency	399.78
14 Spectral Width	10000.0
15 Nucleus	1H
16 Acquired Size	32768
17 Spectral Size	104858

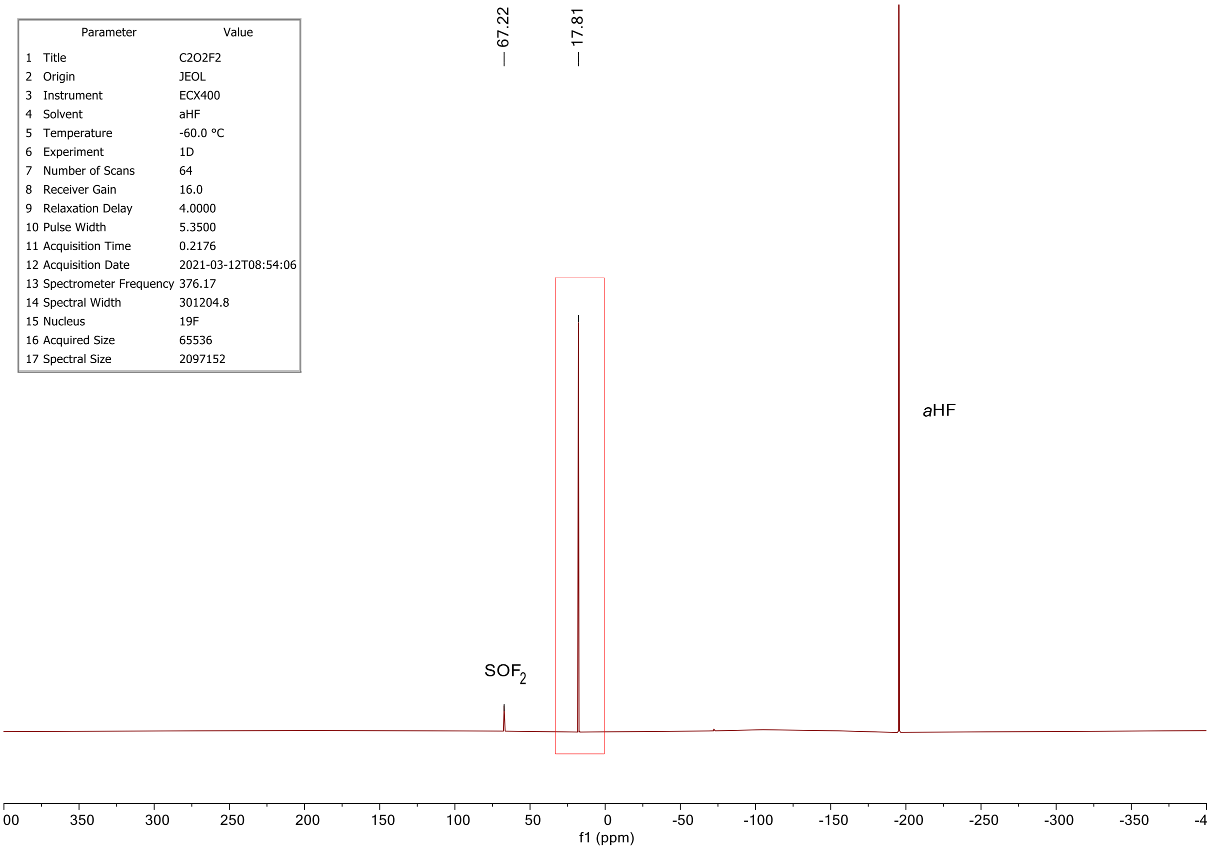

Parameter	Value
1 Title	C2O2F2
2 Origin	JEOL
3 Instrument	ECX400
4 Solvent	aHF
5 Temperature	-60.0 °C
6 Experiment	1D
7 Number of Scans	64
8 Receiver Gain	16.0
9 Relaxation Delay	4.0000
10 Pulse Width	5.3500
11 Acquisition Time	0.2176
12 Acquisition Date	2021-03-12T08:54:06
13 Spectrometer Frequency	376.17
14 Spectral Width	301204.8
15 Nucleus	19F
16 Acquired Size	65536
17 Spectral Size	2097152

Figure S10: ^{1}H and ^{19}F NMR spectra of $C_2O_2F_2$ in *a*HF, −60 °C.

	Parameter	Value
1	Title	C2O2F2
2	Origin	JEOL
3	Instrument	ECX400
4	Solvent	aHF
5	Temperature	-60.0 °C
6	Experiment	1D
7	Number of Scans	3668
8	Receiver Gain	44.0
9	Relaxation Delay	1.0000
10	Pulse Width	2.6667
11	Acquisition Time	0.8651
12	Acquisition Date	2021-03-12T10:51:59
13	Spectrometer Frequency	100.53
14	Spectral Width	30303.0
15	Nucleus	13C
16	Acquired Size	32768
17	Spectral Size	52430

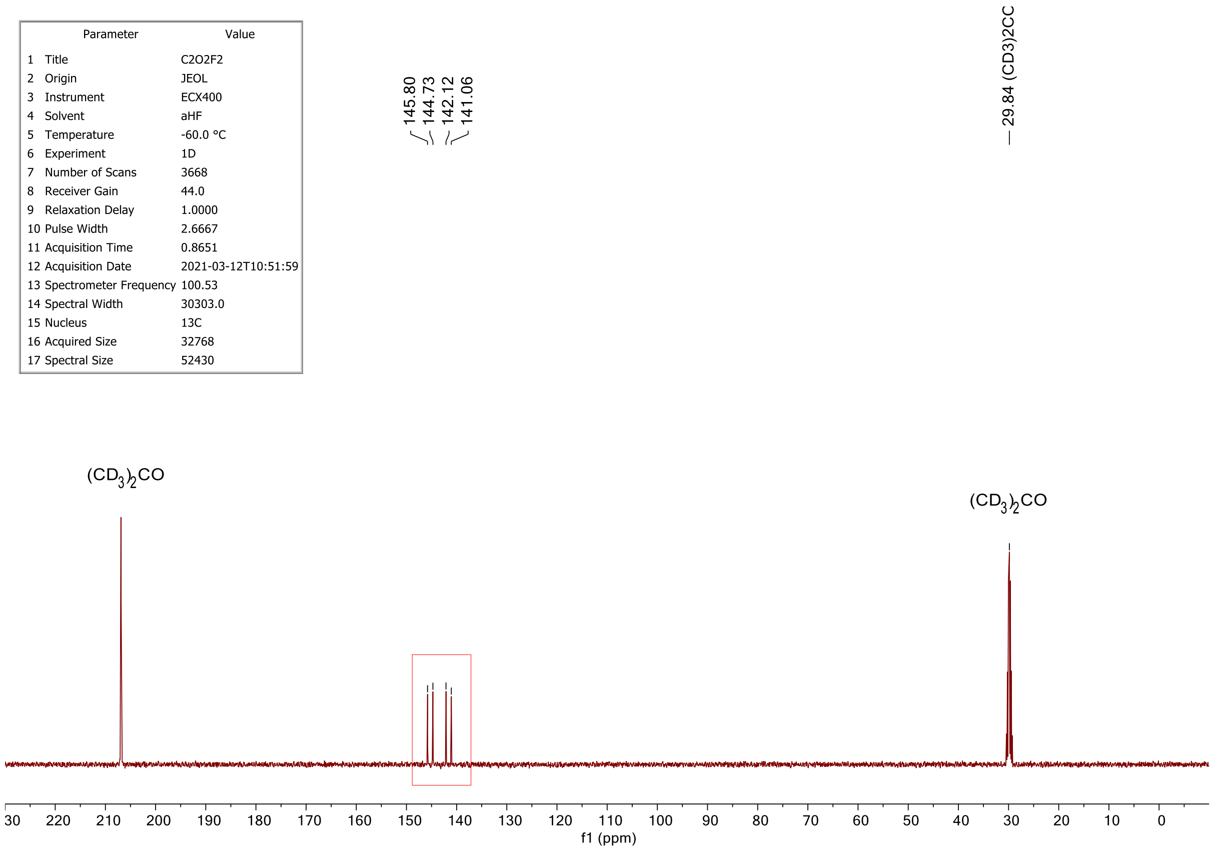

Figure S11: ^{13}C NMR spectrum of $C_2O_2F_2$ in *a*HF, –60 °C.

$C_2O_2F_2$:

^{19}F NMR [377 MHz, –60 °C, $(CD_3)_2CO$)]: δ = 17.81 (s, $C_2O_2F_2$).

^{13}C NMR [101 MHz, –60 °C, $(CD_3)_2CO$)]: δ = 143.4 (dd, *J* = 369.0 Hz, $C_2O_2F_2$).

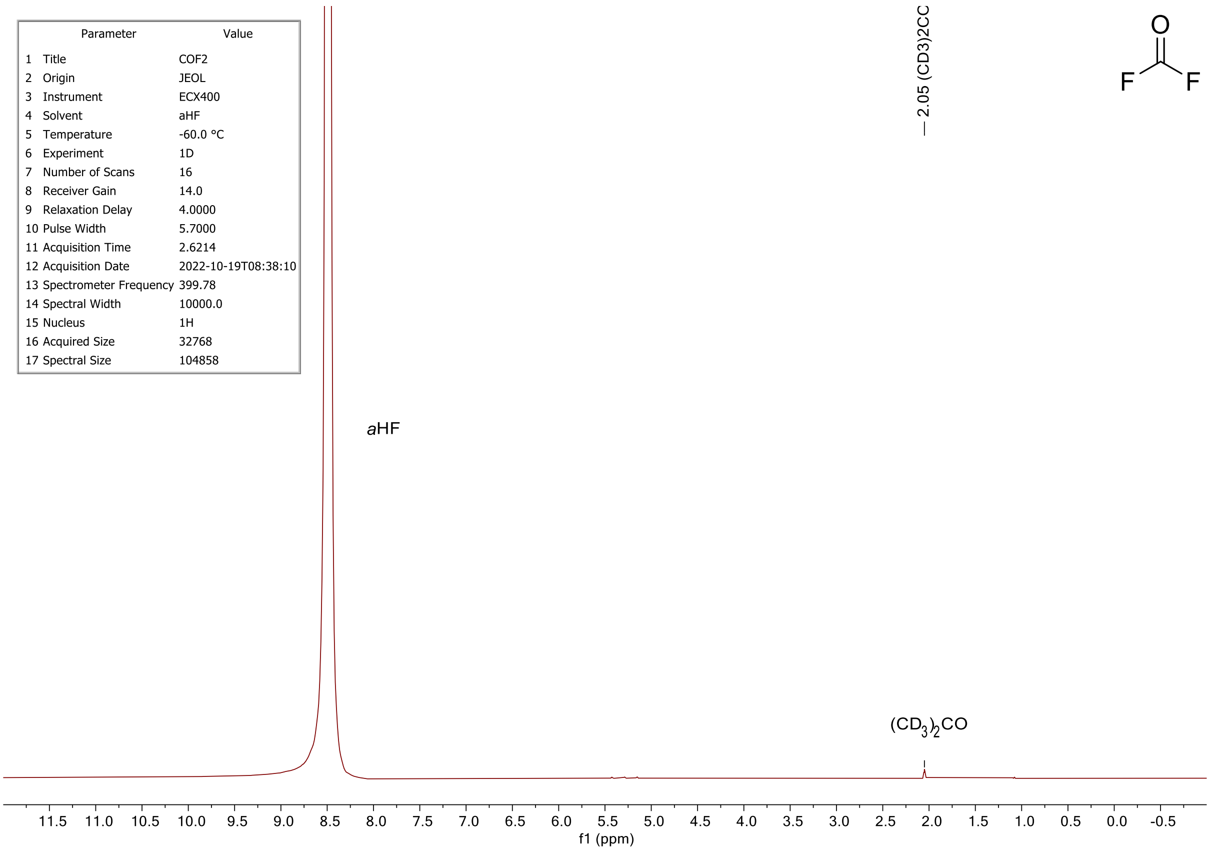

	Parameter	Value
1	Title	COF2
2	Origin	JEOL
3	Instrument	ECX400
4	Solvent	aHF
5	Temperature	-60.0 °C
6	Experiment	1D
7	Number of Scans	16
8	Receiver Gain	14.0
9	Relaxation Delay	4.0000
10	Pulse Width	5.7000
11	Acquisition Time	2.6214
12	Acquisition Date	2022-10-19T08:38:10
13	Spectrometer Frequency	399.78
14	Spectral Width	10000.0
15	Nucleus	1H
16	Acquired Size	32768
17	Spectral Size	104858

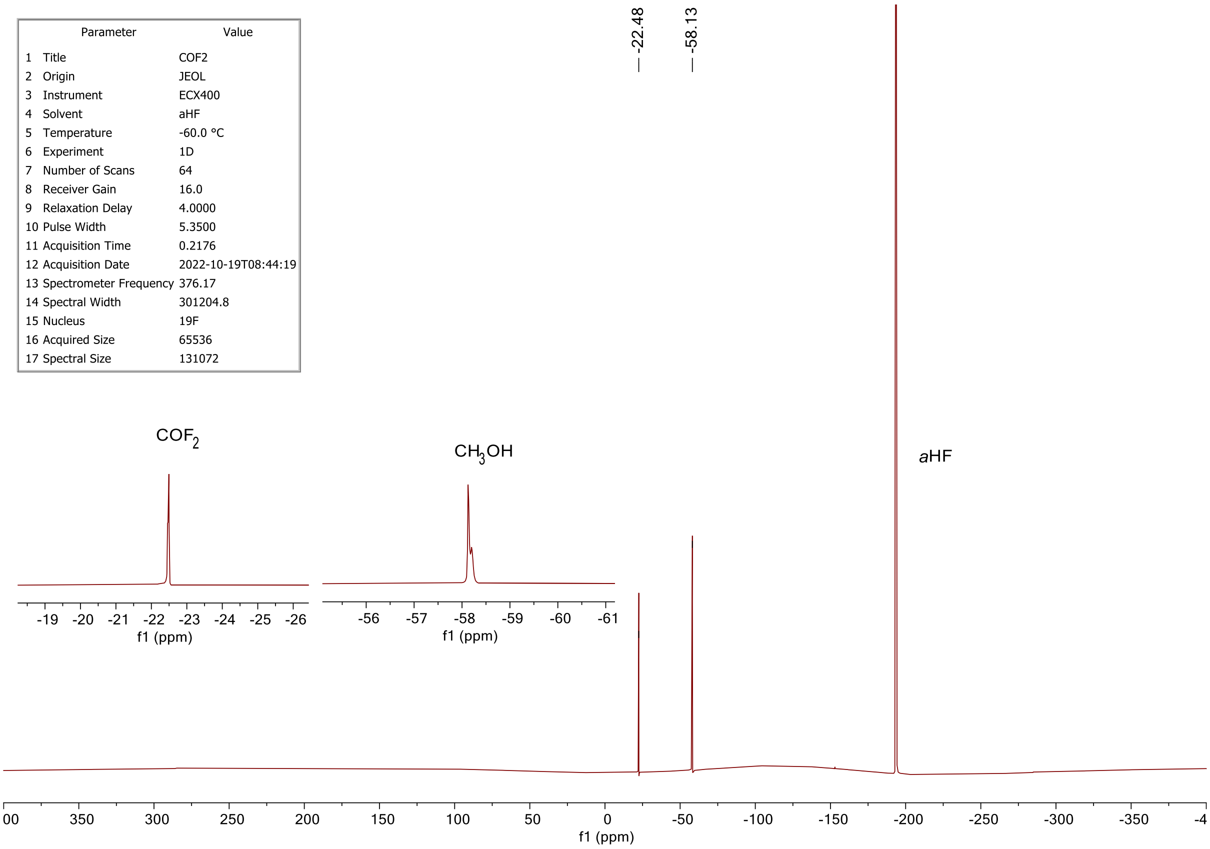

	Parameter	Value
1	Title	COF2
2	Origin	JEOL
3	Instrument	ECX400
4	Solvent	aHF
5	Temperature	-60.0 °C
6	Experiment	1D
7	Number of Scans	64
8	Receiver Gain	16.0
9	Relaxation Delay	4.0000
10	Pulse Width	5.3500
11	Acquisition Time	0.2176
12	Acquisition Date	2022-10-19T08:44:19
13	Spectrometer Frequency	376.17
14	Spectral Width	301204.8
15	Nucleus	19F
16	Acquired Size	65536
17	Spectral Size	131072

Figure S12: ^{1}H and ^{19}F NMR spectra of COF_2 in *a*HF, −60 °C.

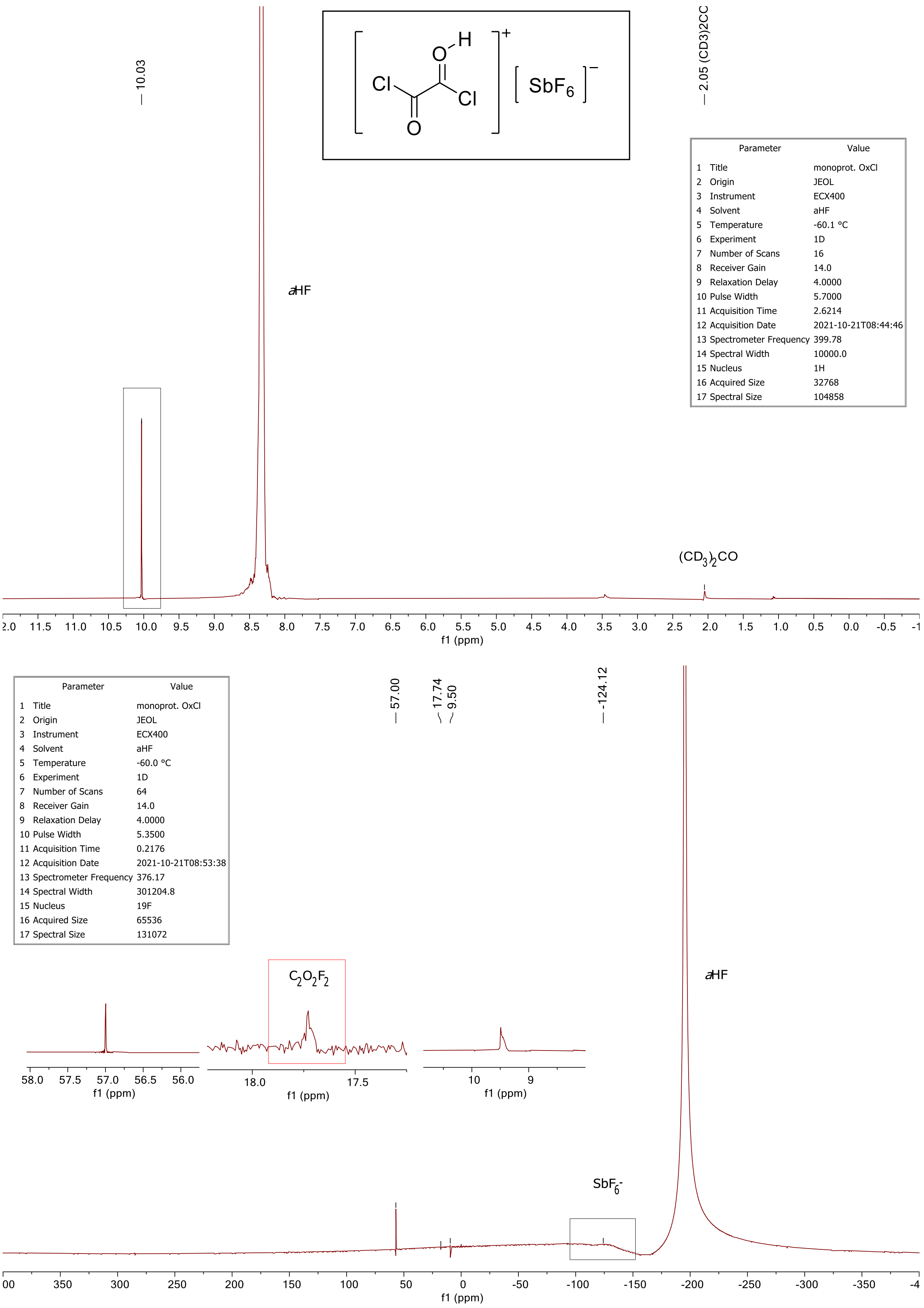

Figure S13: ^{1}H and ^{19}F NMR spectra of $C_2O_2Cl_2$ in HF/SbF_5, −60 °C.

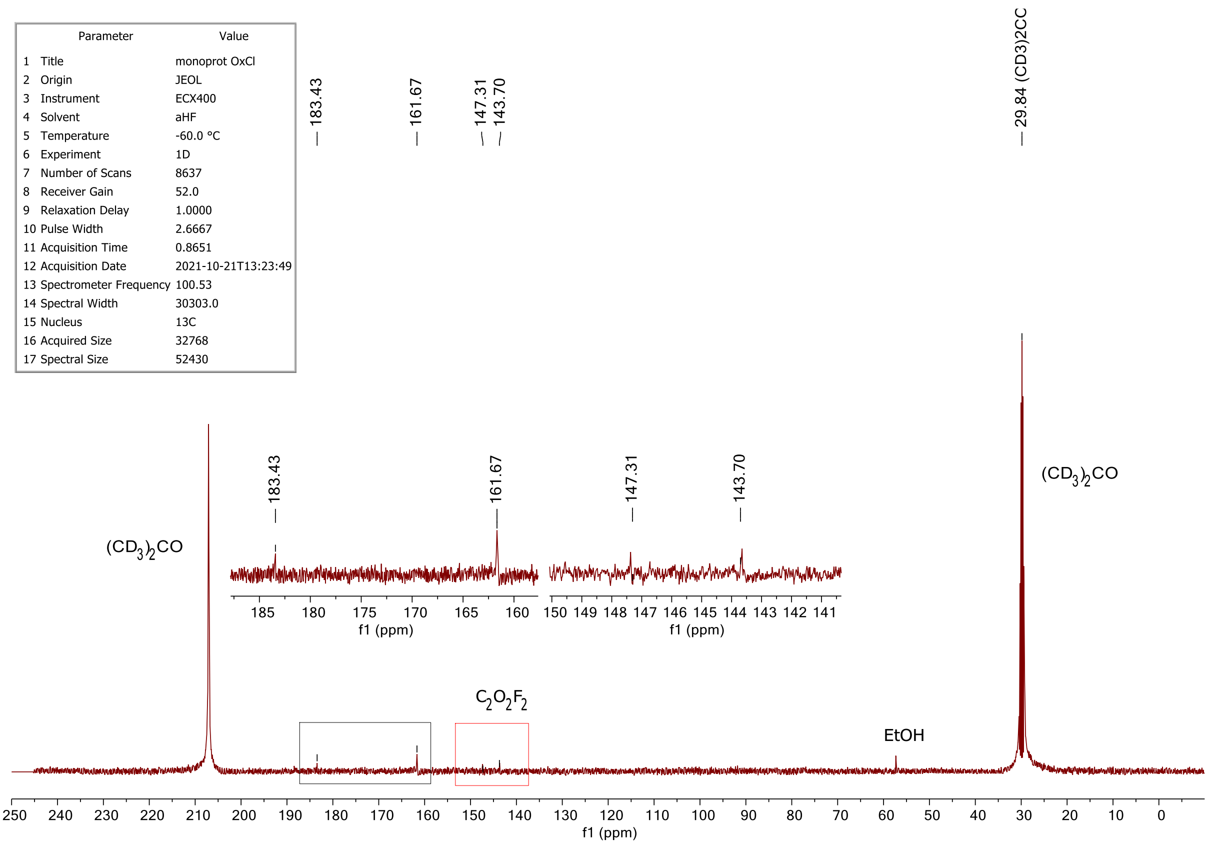

Figure S14: ^{13}C NMR spectrum of $C_2O_2Cl_2$ in HF/SbF_5, –60 °C.

[$C_2O(OH)Cl_2$][SbF_6] (**1**):

^1H NMR [400 MHz, –60 °C, $(CD_3)_2CO$)]: δ = 10.03 (s, COH).

^{19}F NMR [377 MHz, –60 °C, $(CD_3)_2CO$)]: δ = 17.74 (s, $C_2O_2F_2$), –126.14 (s, SbF_6^-).

^{13}C NMR [101 MHz, –60 °C, $(CD_3)_2CO$)]: δ = 183.4 (s, COH), 161.7 (s, CO), 145.0 (d, *J* = 373.8 Hz, $C_2O_2F_2$).

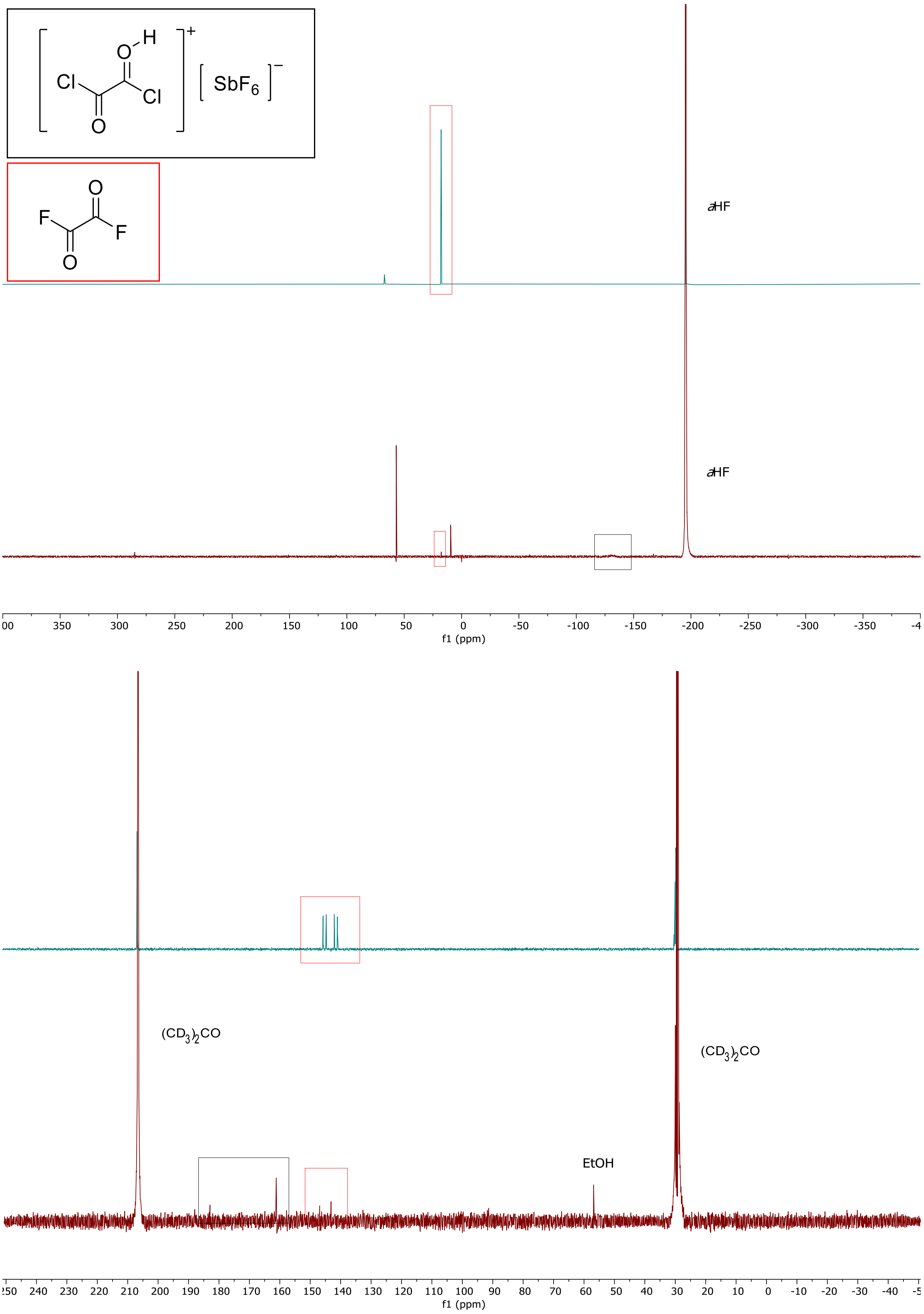

Figure S15: Stacked ^{19}F NMR (top) and ^{13}C NMR (bottom) spectra of $C_2O_2F_2$ and $[C_2O(OH)Cl_2][SbF_6]$ (**1**) in *a*HF, –60 °C.

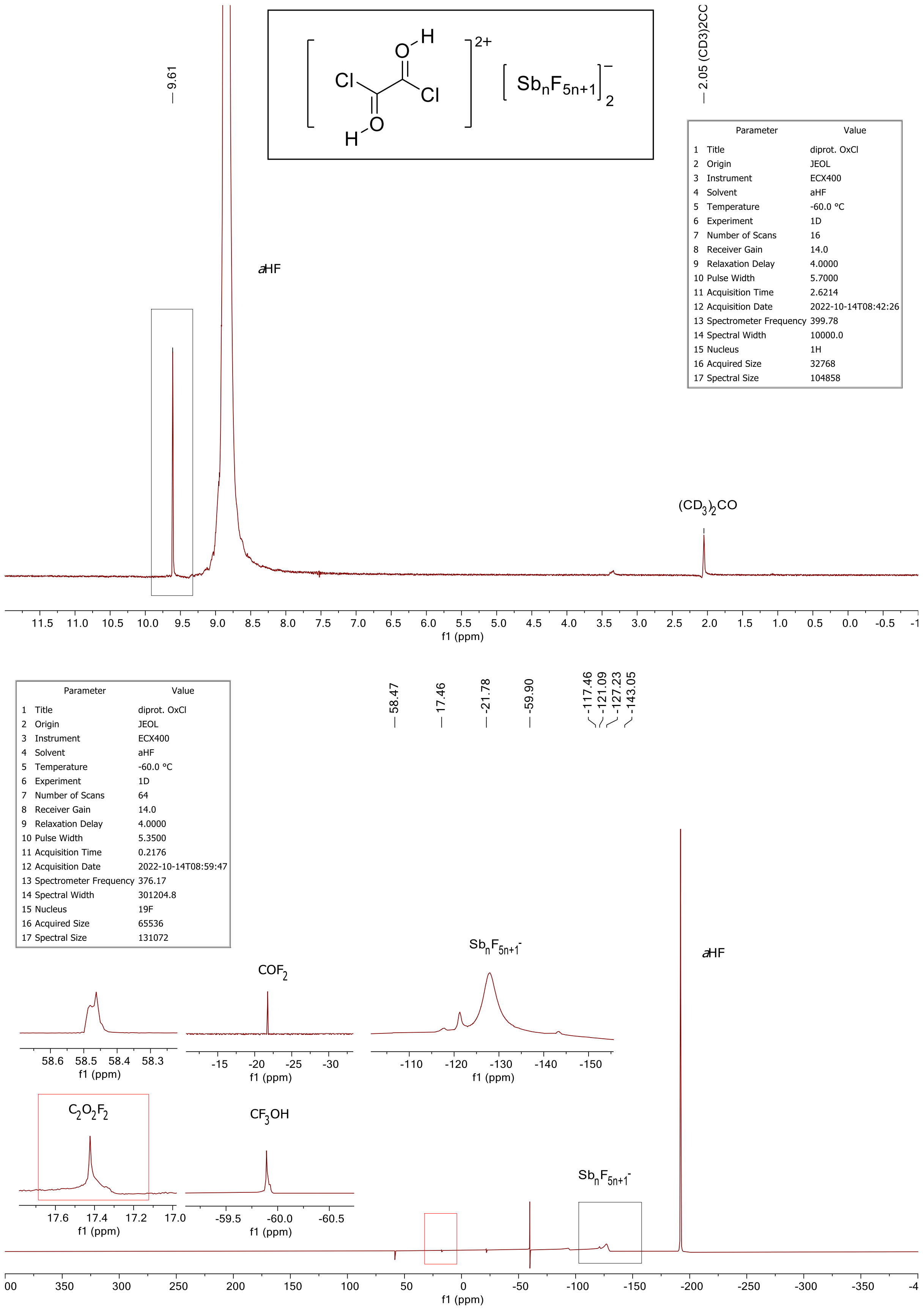

Figure S16: ^{1}H and ^{19}F NMR spectra of $C_2O_2Cl_2$ in HF/8 SbF_5, −60 °C.

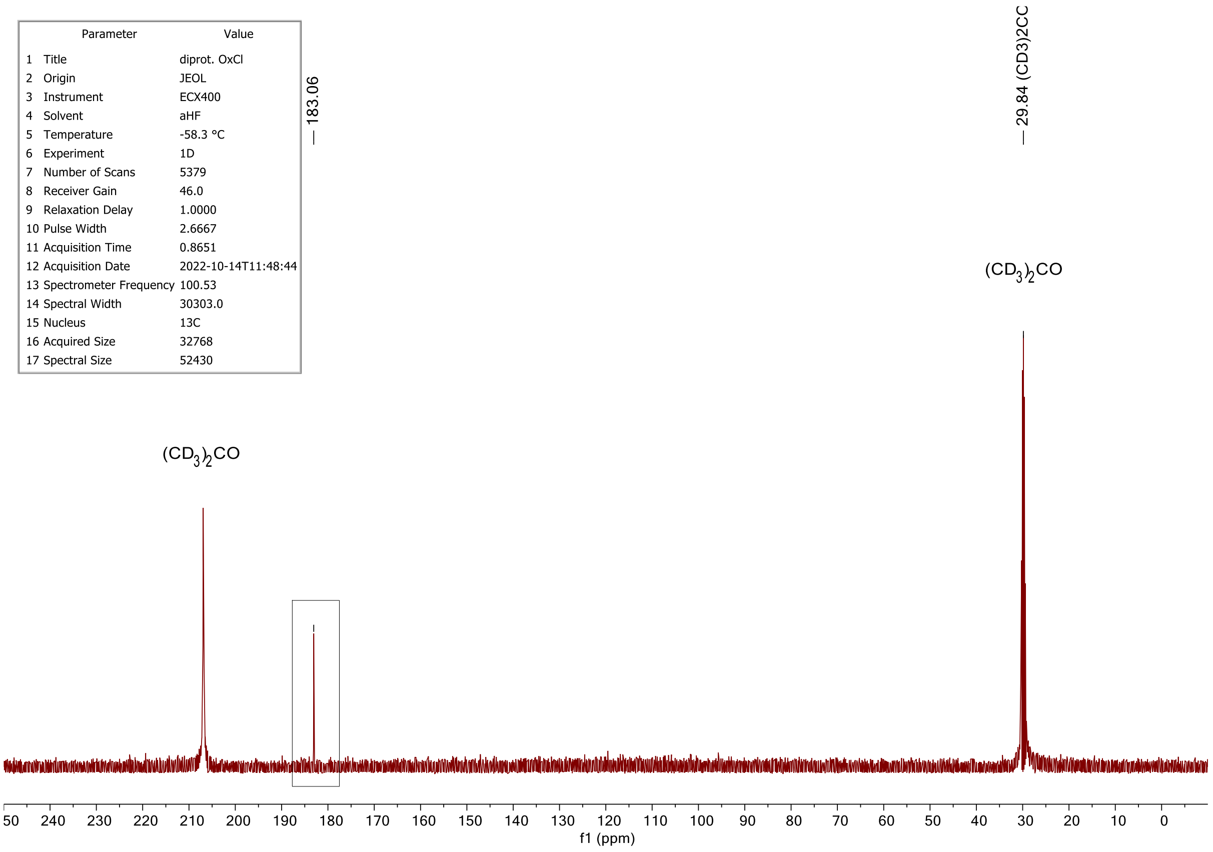

Figure S17: ^{13}C NMR spectrum of $C_2O_2Cl_2$ in HF/8 SbF_5, –60 °C.

$[C_2OH)_2Cl_2][Sb_nF_{5n+1}]$ (**3**):

^{1}H NMR [400 MHz, –60 °C, $(CD_3)_2CO$)]: δ = 9.61 (s, COH).

^{19}F NMR [377 MHz, –60 °C, $(CD_3)_2CO$)]: δ = 17.46 (s, $C_2O_2F_2$), –21.78 (s, COF_2), –59.90 (s, CF_3OH), –117.46, –121.09, –127.23, –143.05 (m, $Sb_nF_{5n+1}^-$).

^{13}C NMR [101 MHz, –60 °C, $(CD_3)_2CO$)]: δ = 183.1 (s, COH).

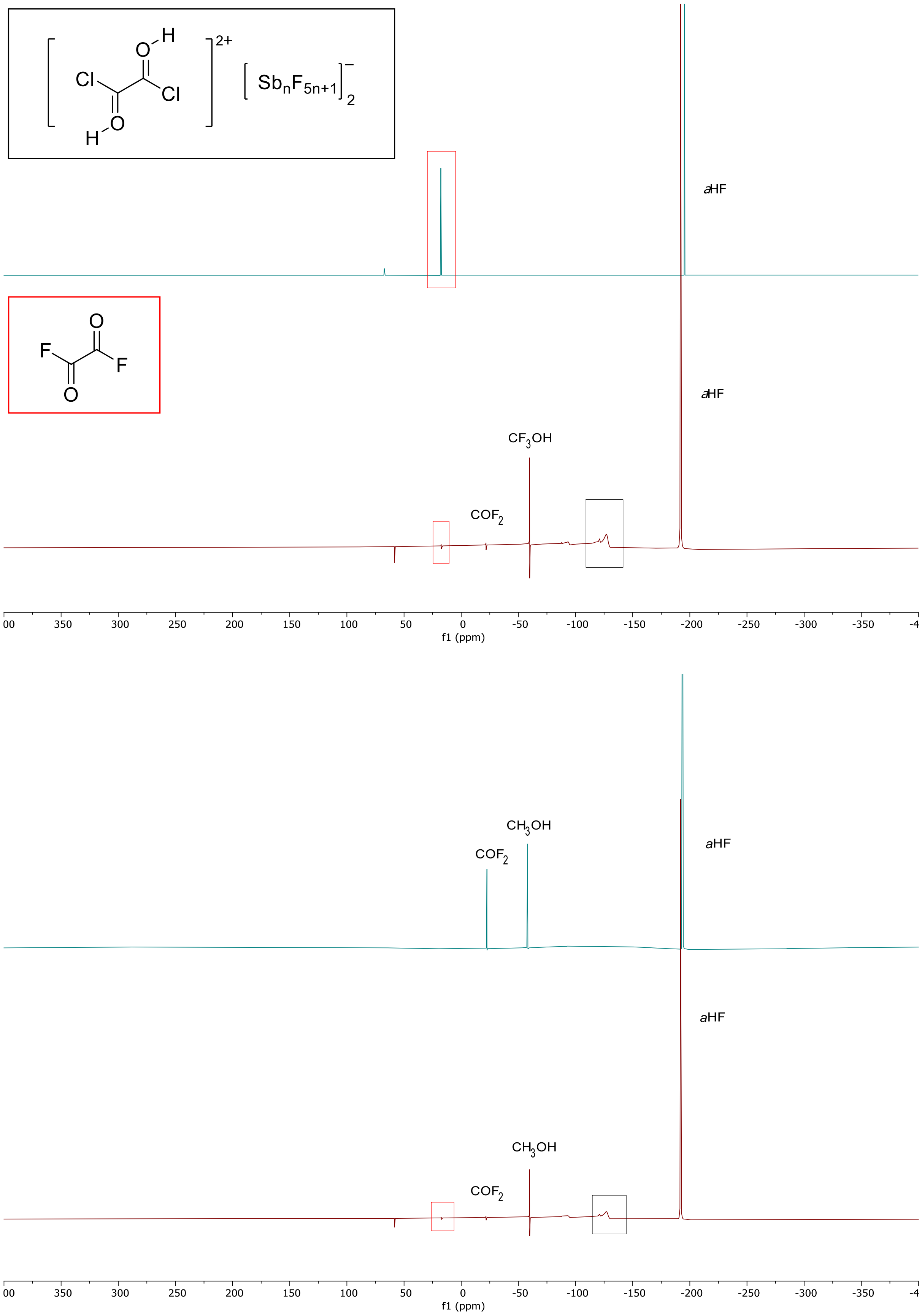

Figure S18: Stacked ^{19}F NMR spectra of $C_2O_2F_2$ (top) and COF_2 (bottom) against $[C_2(OH)_2Cl_2][Sb_nF_{5n+1}]$ (**3**) in *a*HF, −60 °C.

Table S9: Standard orientations of $[C_2O(OH)Cl_2]^+ \cdot HF$. Calculated at the ωB97XD/aug-cc-pVTZ-level of theory.

$[C_2O(OH)Cl_2]^+ \cdot HF$			
Atom	Coordinates [Å]		
	X	Y	Z
F	−3.034538	−1.307737	0.027999
Cl	−1.194106	1.472708	−0.056467
Cl	2.217369	−1.051062	−0.020505
O	−0.631365	−1.019068	−0.041333
H	−1.469943	−1.024178	−0.081766
C	−0.229669	0.138395	−0.026964
O	1.718909	1.476650	0.125000
C	1.301985	0.372434	0.022140
H	−3.748927	−1.099801	0.497905

Table S10: Standard orientations of $[ClCO]^+ \cdot HF$. Calculated at the ωB97XD/aug-cc-pVTZ-level of theory.

$[ClCO]^+ \cdot HF$			
Atom	Coordinates [Å]		
	X	Y	Z
F	−2.661577	0.039057	−0.000459
Cl	−0.103022	−0.062029	0.000113
O	2.569317	−0.006829	−0.000229
C	1.456096	0.121777	0.000279
H	−3.585545	0.026944	0.002377

Table S11: Standard orientations of $C_2O_2Cl_2$. Calculated at the ωB97XD/aug-cc-pVTZ-level of theory.

$C_2O_2Cl_2$			
Atom	Coordinates [Å]		
	X	Y	Z
O	−0.364777	−1.704597	0.000837
C	−0.559192	−0.541966	0.000639
C	0.559105	0.541939	0.000792
O	0.364798	1.704600	0.000771
Cl	−2.170988	0.184054	−0.000627
Cl	2.171009	−0.184046	−0.000635

Table S12: Standard orientations of ClFCO. Calculated at the ωB97XD/aug-cc-pVTZ-level of theory.

ClCOF			
Atom	Coordinates [Å]		
	X	Y	Z
C	−0.000000	0.473396	0.000000
O	−1.026280	1.201626	0.000000
F	1.229285	1.031391	0.000000
Cl	−0.167843	−1.278583	−0.000000

References

[1] Bayersdorfer, L., Minkwitz, R. & Jander, J. (1972). *Z. Anorg. Allg. Chem.* **393**(2), 137-142.

[2] Rigaku OD (2020). *CrysAlis PRO*. Rigaku Oxford Diffraction Ltd, Yarnton, Oxfordshire, England.

[3] Sheldrick, G. M. (2015). *Acta Cryst.* A**71**, 3-8.

[4] Sheldrick, G. M. (2015). *Acta Cryst.* C**71**, 3-8.

[5] Farrugia, L. J. (2012). *J. Appl. Cryst.* **45**, 849-854.

[6] Spek, A. L. (2009). *Acta Cryst.* D**65**, 148-155.

[7] Rigaku OD (2005*). SCALE3 ABSPACK – An Oxford Diffraction Program*, Rigaku Oxford Diffraction Ltd., UK.

[8] Mestrelab Research (2019). *MestReNova 14.0*.

[9] Frisch, M. J., Trucks, G. W., Schlegel, H. B., Scuseria, G. E., Robb, M. A., Cheeseman, J. R., Scalmani, G., Barone, V., Mennucci, B., Petersson, G. A., Nakatsuji, H., Caricato, M., Li, X., Hratchian, H. P., Izmaylov, A. F., Bloino, J., Zheng, G., Sonnenberg, J. L., Hada, M., Ehara, M., Toyota, K., Fukuda, R., Hasegawa, J., Ishida, M., Nakajima, T., Honda, Y., Kitao, O., Nakai, H., Vreven, T., Montgomery, J. A., Peralta, J. E., Ogliaro, F., Bearpark, M., Heyd, J. J., Brothers, E., Kudin, K. N., Staroverov, V. N., Kobayashi, R., Normand, J., Raghavachari, K., Rendell, A., Burant, J. C., Iyengar, S. S., Tomasi, J., Cossi, M., Rega, N., Millam, J. M., Klene, M., Know, J. E., Cross, J. B., Bakken, V., Adamo, C., Jaramillo, J., Gomperts, R., Stratmann, R. E., Yazyev, O., Austin, A. J., Cammi, R., Pomelli, C., Ochterski, J. O., Martin, R. L., Morokuma, K., Zakrzweski, V. G., Voth, G. A., Salvador, P., Dannenberg, J. J., Dapprich, S., Daniels, A. D., Farkas, O., Foresman, J. B., Ortiz, J. V., Cioslowski, J. & Fox, D. J. (2016). *Gaussian16, Revision C.01*, Gaussian Inc., Wallingford CT.

[10] Dennington, R., Keith, T. A. & Millam, J. M. (2016). *GaussView Version 6.0*, Semi-chem Inc. Shawnee Mission, KS.

[11] Bernhardt, E. Willner, H. & Aubke, F. (1999). *Angew. Chem. Int. Ed.* **38**(6), 823-825.

[12] Davis, J. F., Wang, A. & Durig, J. R. (1993). *J. Mol. Struct.* **293**, 27-30.

[13] Nielsen, A. H., Burke, T. G., Woltz, P. J. H. & Jones, E. A. (1952). *J. Chem. Phys.* **20**, 596-604.
